# The COG1-OsSERL2 complex senses cold to trigger signaling network for chilling tolerance in *japonica* rice

**DOI:** 10.1038/s41467-023-38860-4

**Published:** 2023-05-29

**Authors:** Changxuan Xia, Guohua Liang, Kang Chong, Yunyuan Xu

**Affiliations:** 1 The Key Laboratory of Plant Molecular Physiology, Institute of Botany, Chinese Academy of Sciences, Beijing, 100093 China; 2 University of Chinese Academy of Sciences, Beijing, 100049 China; 3 Jiangsu Key Laboratory of Crop Genetics and Physiology/Co-Innovation Centre for Modern Production Technology of Grain Crops, Key Laboratory of Plant Functional Genomics of the Ministry of Education, Yangzhou University, Yangzhou, 225009 China; 4 Present Address: Beijing Vegetable Research Center (BVRC), Beijing Academy of Agricultural and Forestry Sciences, Beijing, 100097 China

**Keywords:** Plant stress responses, Plant signalling, Plant domestication

## Abstract

Improvement of chilling tolerance is a key strategy to face potential menace from abnormal temperature in rice production, which depends on the signaling network triggered by receptors. However, little is known about the QTL genes encoding membrane complexes for sensing cold. Here, *Chilling-tolerance in* *Gengdao*/*japonica rice* *1* (*COG1*) is isolated from a chromosome segment substitution line containing a QTL (*qCS11-jap*) for chilling sensitivity. The major gene *COG1* is found to confer chilling tolerance in *japonica* rice. In natural rice populations, only the haplogroup1 encodes a functional COG1. Evolutionary analysis show that *COG1* originates from Chinese *O. Rufipogon* and is fixed in *japonica* rice during domestication. COG1, a membrane-localized LRR-RLP, targets and activates the kinase OsSERL2 in a cold-induced manner, promoting chilling tolerance. Furthermore, the cold signal transmitted by COG1-OsSERL2 activates OsMAPK3 in the cytoplasm. Our findings reveal a cold-sensing complex, which mediates signaling network for the chilling defense in rice.

## Abstract (web summary)

Improvement of chilling tolerance is a key strategy in rice production. Here the authors report that the QTL gene *COG1* confers chilling tolerance in *japonica* rice. COG1 interacts and activates OsSERL2 to transmit the cold signal for chilling defense.

## Introduction

Rice (*Oryza sativa* L.) is a major food crop that feeds half of the world’s population. But temperature stress (such as chilling) threatens rice production^1,2^; low temperature is one of the major environmental factors that limits the geographic distribution of rice cultivation^2–4^. And temperature fluctuations have become increasingly intense and frequent caused by the global climate change^5,6^. Evolutionarily, cultivated rice originated from wild accessions in the subtropical regions of Asia, and subsequently expanded to a wide latitudinal range^7^. Domesticated rice, including the major subspecies *japonica* and *indica*, are characterized by specific climate adaptations based on agro-ecological cultivation conditions^8^. Therefore, molecular genetic tools have been urgently sought to improve chilling tolerance of rice to fight climate change and expand it into northern areas with relatively low yearly temperatures.

Chilling tolerance during rice growth and development is a quantitative trait controlled by multiple genetic factors. Delineation of the genetic basis underlying chilling tolerance in rice is still in its infancy. A number of quantitative trait loci (QTLs) that confer chilling tolerance have been mapped, and some major genes have been genetically characterized^2–4,9,10^. But less is known about the molecular basis underlying the divergence of environmental adaptations and geographical distribution between *japonica* and *indica* varieties.

Receptors on the plasma membrane recognize exogenous and endogenous signals to trigger appropriate cellular responses during plant growth and development. Leucine-rich repeat receptor like proteins (LRR-RLPs) are a plant membrane protein family, members of which have an extracellular LRR domain but lack an intracellular kinase domain^11^. LRR-RLPs reportedly function in plant development. For example, AtRLP17/TOO MANY MOUTHS, AtRLP10/CLAVATA2, and AtRLP44 participate in stomatal development, maintenance of the apical meristem, and cell wall perturbations, respectively, in Arabidopsis^12–14^. Other members of LRR-RLPs were reported to be involved in Arabidopsis immune response such as AtRLP1, AtRLP23, AtRL30, AtRLP42, and AtRLP51^15–19^. In rice, OsRLP1 associates with its adapter kinase OsSOBIR1 to mediate plant immunity against viral infection^20^. AtLRRop2 is involved in either the chilling or freezing stress response in Arabidopsis^21^. However, it is unknown how such LRR-RLPs sense environmental cold for the tolerance in rice.

In the absence of a kinase domain, LRR-RLP likely acts as a receptor or regulator, transmitting signals by forming complexes with somatic embryogenesis receptor kinases (SERKs) and other transmembrane or membrane associated proteins^22,23^. SERKs are a subgroup of leucine-rich repeat receptor-like kinases (LRR-RLKs) that operate as co-receptors of various RLKs and RLPs to regulate immunity, growth, and development^23^. In Arabidopsis, AtSERK3/BRI1-ASSOCIATED RECEPTOR KINASE 1 not only acts as a co-receptor of BRI1 and FLS2, affecting the brassinosteroid and immune signaling pathway, respectively^24,25^; but also acts as a co-receptor of AtRLP17 in stomatal development, of AtRLP44 in cell wall integrity regulation and of AtRLP23 in immune activation^12–14^. In addition, AtSERK3 homologs, namely AtCIKs, act as co-receptors for AtRLP10^26^. In tomato, S1SERK3 is required for the RLP proteins Cf-9, Cf-2, CF-4, Cf-5, LeEIX1/2, and Ve1 to mediate immune activation^22,27^. The rice genome contains two *SERK* and seven SERK-like (*SERL*) genes that have roles in rice development^28^ (http://rice.uga.edu/). However, it is unclear whether SERKs/SERLs form complexes with LRR-RLPs to transmit signals in rice.

Cold temperature triggers plasma membrane rigidification, Ca^2+^ channel or activation of RLKs/RLPs in plant cells. This signaling subsequently leads to the activation of kinase in the cytoplasm^2,29,30^. For example, cold stress activates kinase activity of the plasma membrane-localized proteins CRPK1 and CPK28 to promote phosphorylation of 14-3-3 proteins and NLP7, respectively, in the cytosol^31,32^. Receptor like kinases CRLK1 and CRLK2 activate the MEKK1-MKK1/2-MPK4 cascade under cold stress to enhance freezing tolerance in Arabidopsis^33^. Similarly, unknown activators in the plasma membrane rapidly activate the MKK4/5-MPK3/6 cascade in response to cold stress, negatively regulating cold tolerance^34^. In rice, the cold-activated OsMAPK3 phosphorylates the transcription factor OsbHLH002 to enhance chilling tolerance^35^. Another cytoplasmic kinase, OsSAPK6, phosphorylates IPA1 to activate chilling tolerance responses^36^. However, the identities of specific plasma membrane activators, such as LRR-RLP/RLK complexes, that activate kinases in the cytoplasm to respond to chilling signals for tolerance in rice are unknown.

Here, we characterized *Chilling-tolerance in* *Gengdao/japonica rice* *1* (*COG1*), a major gene identified in a QTL for positive regulation of chilling tolerance in *japonica* rice. Our data suggested that a functional *COG1* allele originated from Chinese populations of *O. rufipogon* and was under selection during *japonica* rice domestication. COG1 was determined to be an LRR-RLP and to form a complex with OsSERL2, regulating activation of the latter for sensing cold signals in the membrane. Furthermore, the COG1-OsSERL2 complex activated OsMAPK3 to modulate chilling tolerance. These findings are significant not only in shedding light on the cold signals network but also in the potential for the genetic improvement for the trait in rice breeding.

## Results

### Map-based cloning of QTLs from chromosome segment substitution lines

**Fig. 1:**
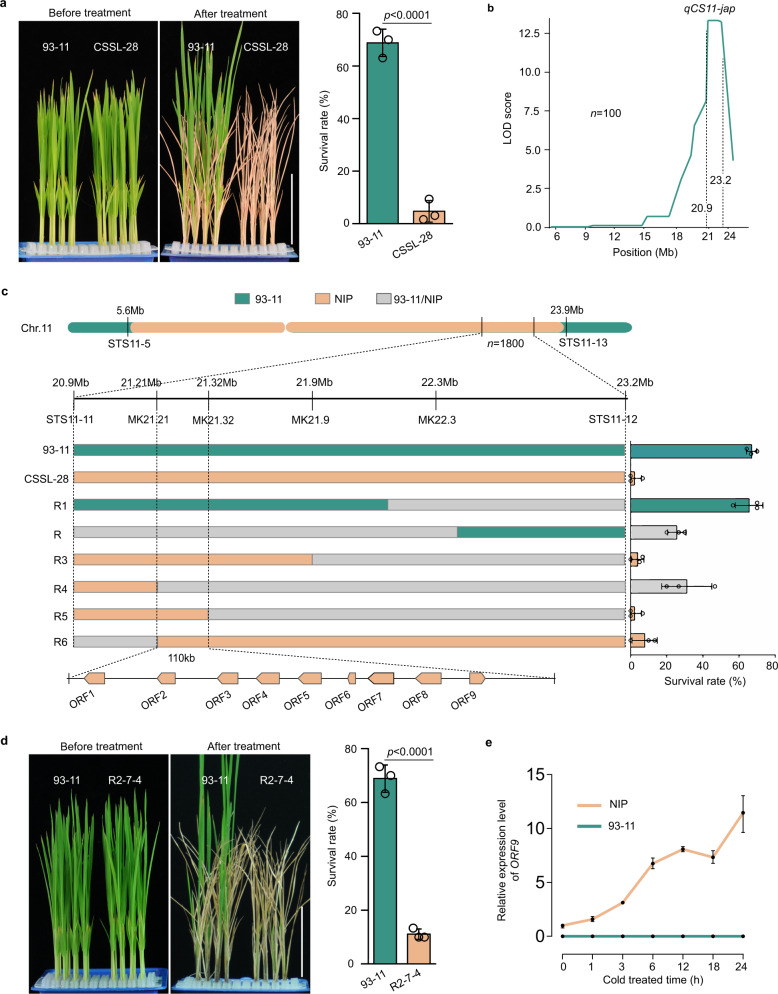
Map-based cloning of the major gene in *qCS11-jap*. **a** Chilling tolerance of 93-11 (*indica*) and of chromosome segment substitution line 28 (CSSL-28). The survival rates were determined after a 4-week recovery period. Data are means ± SD (two-tailed Student’s *t*-test; *n* = 3 biological replicates). Scale bars = 6 cm. **b** Location of *qCS11-jap* on chromosome 11 revealing by LOD score plots. **c** Fine mapping of the *qCS11-jap* locus. Left, high resolution mapping. STS11-5, STS11-11, MK21.21, MK21.32, MK21.9, MK22.3, STS11-12 and STS11-13 were molecular markers (Supplementary Data 5). Right, survival rates of six recombinants (R1–R6) and the parents. Data are means ± SD (n = 3 biological replicates). **d** Chilling tolerance of 93-11 and a homozygous recombinant line (R2-7-4). Data are means ± SD (two-tailed Student’s *t*-test; *n* = 3 biological replicates). Scale bars = 6 cm. **e** The expression levels of *ORF9* (*LOC_Os11g36200*) in 93-11 and Nipponbare (NIP) with different treatment times at 4 °C. Data are means ± SD (*n* = 3 biological replicates). See also Supplementary Fig. 1. Source data are provided as a Source Data file.

In order to elucidate the genetic basis for rice chilling tolerance divergence, a wide population consisting of 128 chromosome segment substitution lines (CSSLs) was generated by crossing and back-crossing using the chilling-sensitive *indica* variety 93-11 as the recurrent parent and the chilling-tolerant *japonica* variety Nipponbare as the donor parent^37^. Then, 72 of 128 individual lines were randomly selected from the CSSLs population to test their cold tolerance. After the seedlings were treated at 4 °C for 44 h, the survival rates of all selected lines ranged from 60–95%, except for CSSL-28, this line had a survival rate of ~5% (Fig. 1a and Supplementary Fig. 1a). The CSSL-28 was selected for further analysis due to its chilling sensitivity. Molecular markers assay showed that the substituted chromosome segment from Nipponbare was located between 5.6 and 23.9 Mb on chromosome 11 in CSSL-28. These results suggested that the *japonica* insertion of the CSSL-28 may involve a putative quantitative trait locus from *japonica* rice conferring Chilling Sensitivity on *indica* rice in chromosome 11 (*qCS11-jap*).

To clone the major genes underlying *qCS11-jap*, CSSL-28 was backcrossed with 93-11 then self-crossed to obtain a F_2:3_ population. Hundred individuals of the F_2:3_ population were used to detect the chilling tolerance and were genotyped using nine markers from the 5.6–23.9 Mb region (Supplementary Data 1). LOD score plots revealed that *qCS11-jap* was within the 2.3-Mb interval between STS11-11 (20.9 Mb) and STS11-12 (23.2 Mb) (Fig. 1b).

All 1800 individuals comprising the F_2:3_ population were analyzed, and six recombinants (R1–R6) with recombination between STS11-11 and STS11-12 were selected. The cold tolerance and genotypes of these six recombinants, 93-11 and CSSL-28 were tested to isolate the major gene underlying *qCS11-jap*. The results showed that the survival rate of R1 without Nipponbare segment in the STS-11-11–MK22.3 region was not significantly different compared with 93-11; the survival rate of R2 with heterozygous segment fell between the rates of 93-11 and CSSL-28. These results indicated that *qCS11-jap* was located in the region between STS-11-11 and MK22.3. The survival rate of R3 was closer to that of CSSL-28, indicating that *qCS11-jap* could be further mapped in the region between STS-11-11 and MK21.9. R4–R6 were chilling-sensitive, showing survival rates comparable to that of CSSL-28. The relevant region of *qCS11-jap* was thus narrowed to the region between MK21.21 and MK21.32 (Fig. 1c). Sequence analysis showed that there are 9 predicted genes (*ORF1*–*ORF9*) in the 110-kb mapping region (Fig. 1c).

To determine the major gene in this region, a homozygous recombinant line (R2-7-4) was selected from the offspring of the F_2:3_ population using molecular markers. R2-7-4 contained the Nipponbare segment from 21.2–22.6 Mb (Supplementary Fig. 1b). Chilling tolerance test showed that the survival rate of R2-7-4 (15.0%) was significantly lower than that of 93-11 (68.7%) (Fig. 1d). The expression levels of all 9 predicted genes (Fig. 1c) within this region in 93-11 and R2-7-4 were detected using transcriptome sequencing after chilling treatment for 0, 4, 24 h. The results showed that the expression of *ORF9* was clearly induced by chilling in R2-7-4 (Supplementary Fig. 1c). To further validate the transcriptome data, the transcript levels of *ORF9* in the chilling-treated seedlings of 93-11 and Nipponbare were detected by qRT-PCR. The results showed that *ORF9* was consistently induced by chilling in Nipponbare but not expressed in 93-11 (Fig. 1e). Therefore, *ORF9* (*LOC_Os11g36200*) may be the candidate gene for response to the chilling.

### *COG1* is essential for chilling tolerance in *japonica* rice

**Fig. 2:**
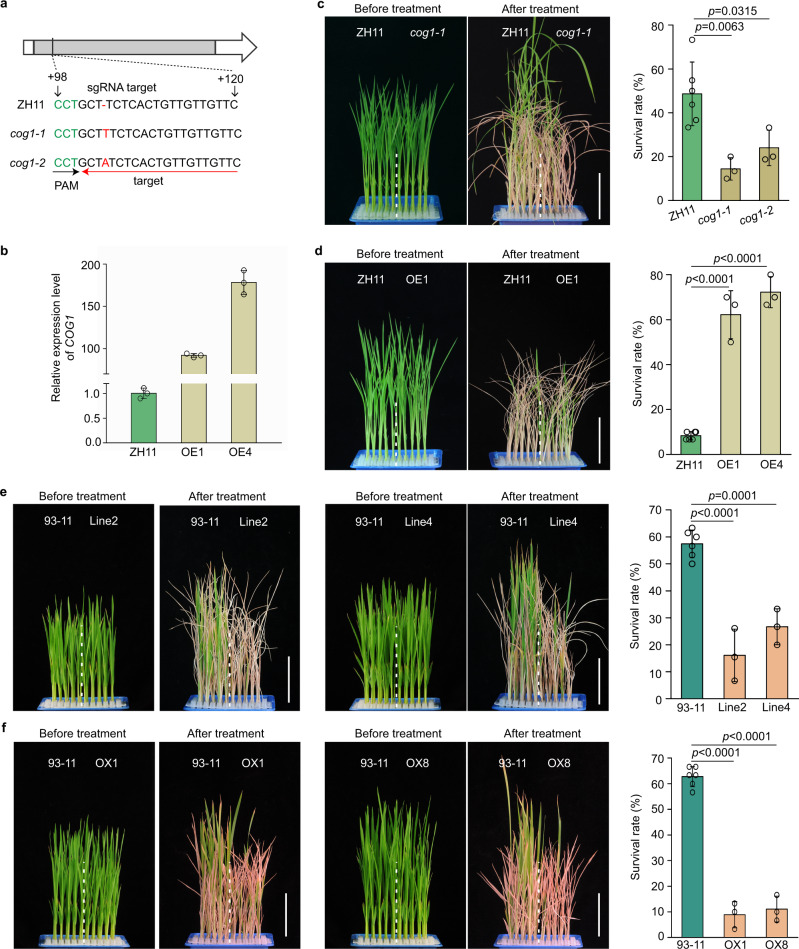
*COG1* is essential for chilling tolerance in *japonica* rice. **a** Identification of *cog1-1* and *cog1-2* via sequencing. PAM, protospacer adjacent motif. **b** The expression levels of *COG1* in ZH11 and two *COG1-*overexpression lines (OE1 and OE4). Data are means ± SD (*n* = 3 biological replicates). **c**, **d** The chilling tolerance of *COG1* mutants (*cog1-1* and *cog1-2*) (**c**) and *COG1-*overexpression lines (**d**) in the ZH11 background. The seedlings of mutants and overexpressed lines were treated at 4 °C for 72 h and 80 h, respectively. The survival rates were determined after a 4-week recovery period. Data are shown as the means of three replicates ± SD (two-tailed Student’s *t*-test). Bar = 6 cm. **e** The chilling tolerance of *COG1::COG1-HA* transgenic lines (Line2 and Line4) in the *indica* 93-11 background. The seedlings were treated at 4 °C for 44 h. Bar = 6 cm. Data are means ± SD (two-tailed Student’s *t*-test; *n* = 3 biological replicates). **f** The chilling tolerance of *COG1-*overexpression lines (OX1 and OX8) in the 93-11 background. The seedlings were treated at 4 °C for 44 h. Bar = 6 cm. Data are means ± SD (two-tailed Student’s *t*-test; *n* = 3 biological replicates). See also Supplementary Fig. 2. Source data are provided as a Source Data file.

To determine whether *ORF9*, here designated *Chilling-tolerance in* *Gengdao*/*japonica rice* *1* (*COG1*) underlies the QTL, two loss-of-function mutants (*cog1-1* and *cog1-2*), mutated by the CRISPR/cas9 approach, and two overexpression lines (OE1 and OE4) were generated in the *japonica* cultivar Zhonghua 11 (ZH11) (Fig. 2a, b and Supplementary Fig. 2a). The survival rate of ZH11 was 49% after the chilling treatment for 72 h, while those of *cog1-1* and *cog1-2* were 15% and 26%, respectively (Fig. 2c). In contrast, the OE1 and OE2 exhibited higher survival rates (62% and 72%, respectively) than that of ZH11 (9%) when the seedlings were treated for 80 h by cold (Fig. 2d). In the *indica* background, on the contrary, both the overexpression lines and the *COG1::COG1-HA* transgenic lines showed decreased chilling tolerance compared with the wild type 93-11, confirming the chilling-sensitive phenotype of CSSL-28 (Figs. 2e, f and Supplementary Fig. 2b, c). The genomic sequence analysis data showed that *COG1* was disrupted by a 6,665-bp insertion in the *indica* 93-11 (Supplementary Fig. 2d), consistent with the qRT-PCR results (Fig. 1e). This difference between the phenotypes of *COG1*-overexpression lines in *japonica* and *indica* rice may result from differences in their genetic backgrounds. Such similar case is also seen in other genes^38–40^. Therefore, these results suggest that *COG1* positively modulates chilling tolerance in *japonica* rice, but its invading in *indica* rice showed a negative regulator.

### *COG1* is selected during *japonica* rice domestication

**Fig. 3:**
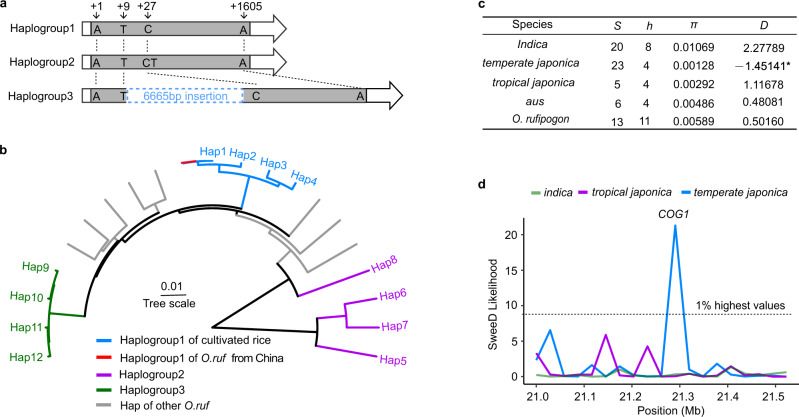
*COG1* is selected during *japonica* rice domestication. **a** The three *COG1* haplogroups identified in 67 representative rice accessions. The Nipponbare genome sequence was used as a reference. **b** Phylogenetic tree showing the relationships of *COG1* haplotypes. Phylogenetic tree was constructed using the sequences of *COG1* in 67 representative rice accessions. **c** Nucleotide polymorphism and neutrality tests for *COG1* in the 67 representative rice accessions. *S*: Number of sample. *h*: Number of haplotypes. π: Average number of pairwise nucleotide differences per site calculated on the silent sites. *D*: Tajima’s *D* value. **d** The within population selection signal value (SweeD) of the 500-kb region flanking *COG1*. See also Supplementary Data 2 and 3.

Sequence comparison between Nipponbare and 93-11 indicated that *COG1* of Nipponbare is a 1605-bp open reading frame encoding 534 amino acids, but *COG1* of 93-11 contain a 6,665-bp insertion at position +9 from the start codon, resulting in silence of this gene (Fig. 3a and Fig. 1e). Variation of *COG1* sequences were then investigated in natural populations using a pan-genome dataset comprising 67 accessions that represent all major genetic clusters in *O. sativa* and *O. rufipogon*^41^, including 23 accessions of *O. sativa temperate japonica*, 5 of *O. sativa tropical japonica*, 20 of *O. sativa indica*, 6 of *O. sativa aus* and 13 of *O. rufipogon* (Supplementary Data 2). Analysis of the *COG1* coding region revealed 12 haplotypes, which could be divided into three groups (haplogroup1 to 3) (Fig. 3a, Supplementary Data 2 and 3). All of the selected 28 *japonica* rice accessions (including Nipponbare) and 3 *O. rufipogon* varieties (which originated from China) belonged to haplogroup1, which encoded a full-length COG1 protein. In contrast, the haplogroup2 had a 1-bp insertion (T) at position +28 resulting in a frameshift in 9 *indica* and 3 *aus* accessions. In addition, the haplogroup3, containing a 6,665-bp insertion at +9, was found in 8 *indica* (including 93-11) and 2 *aus* accessions (Fig. 3a, Supplementary Data 3). Phylogenetic analysis based on the *COG1* sequences of the 67 accessions indicated that clustering among all *japonica* accessions and the Chinese *O. rufipogon* accessions carrying the chilling-tolerant haplogroup1 (Fig. 3b and Supplementary Data 3). These observations suggested that the haplogroup1 of *COG1* originated from Chinese *O. rufipogon* and was fixed in *japonica* rice during domestication.

To examine whether domestication has acted on *COG1*, nucleotide diversity was analyzed in the *COG1* sequences of the 67 representative rice accessions. A comparison of the nucleotide diversity among *indica*, *tropical japonica*, *temperate japonica*, *aus* and *O. rufipogon* accessions indicated that *temperate japonica* (*π* = 0.00128) exhibited lower diversity than *indica* (*π* = 0.01069), *tropical japonica* (*π* = 0.00292), *aus* (*π* = 0.00486) and *O. rufipogon* (*π* = 0.00589) (Fig. 3c). Tajima’s *D*^42^ values were calculated for each accession, and significantly negative values were observed only in *temperate japonica* accessions (Fig. 3c), indicating that natural selection or genetic bottleneck played a critical role during the northward expansion of rice planting. To further determine this result, SweeD scanning was performed using the resequencing data of the 1176 landraces in the 3000 Rice Genomes Project^8,43,44^. The result showed that a selection signal was detected only in *temperate japonica* landraces in the 500-kb region flanking *COG1* (Fig. 3d), suggesting that *COG1* had been subject to human artificial selection. Taken together, the *COG1* conferring chilling tolerance in *japonica* rice might represent an ancient allele preserved in Chinese populations of *O. rufipogon* and selected during domestication of *japonica* rice.

### *COG1* encodes a plasma membrane and ER localized LRR-RLP

**Fig. 4:**
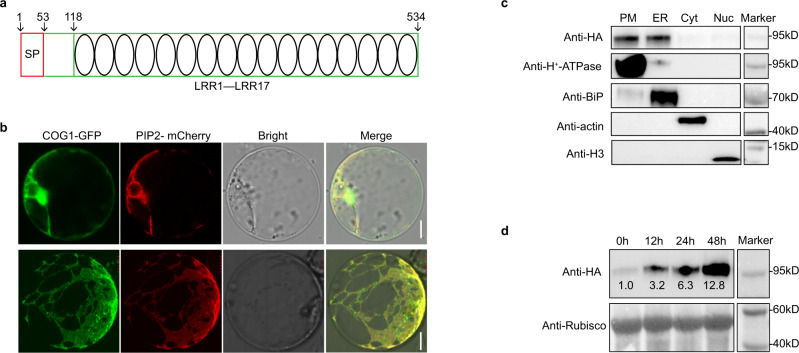
*COG1* encodes a plasma membrane and ER localized LRR-RLP. **a** Schematic of COG1 protein structure. Amino acid residues 1 to 53 comprise the signal peptide (SP), and residues 118 to 534 form 17 LRR domains. See also Supplementary Fig. 3a. **b** Localization of COG1 in rice protoplasts, plasma membrane (upper panel) and ER (down panel). PIP2-mCherry is a marker for the plasma membrane and ER. Scale bar = 10 μm. **c** Localization of COG1 revealing by immunoblotting assay. The COG1-HA fusion protein in *COG1::COG1-HA* transgenic rice (*COG1-HA*-L1) in the ZH11 background was detected by anti-HA antibody. H^+^-ATPase, BiP, actin, and histone 3 (H3) were used as markers of the plasma membrane protein (PM), ER protein, cytoplasmic protein (Cyt), and nuclear protein (Nuc), respectively. **d** COG1 accumulation in *COG1-HA*-L1 exposed to chilling treatment for 0, 12, 24, and 48 h. The ratio of the intensity of the COG1-HA band to that of the rubisco band at 0 h was set as 1.0. Similar results of b–d were observed from three independent biological repeats. Source data are provided as a Source Data file.

*COG1* was predicted to encode an LRR-RLP with 17 LRRs based on its DNA sequences (Fig. 4a and Supplementary Fig. 3). In order to analyze the subcellular localization, COG1-GFP was expressed in rice protoplasts. The confocal observation showed that fluorescence of COG1-GFP overlapped with that of PIP2-mCherry, a marker for the plasma membrane and endoplasmic reticulum (ER)^45^ (Fig. 4b). To confirm this subcellular localization, the proteins of plasma membrane, ER, cytoplasmic, and nuclear fractions were extracted from the *COG1::COG1-HA* transgenic rice in the ZH11 background (*COG1-HA*-L1) and detected by anti-HA antibody. The immunoblotting results showed that COG1-HA was clearly enriched in the plasma membrane and ER fractions (Fig. 4c). To analyze the effects of chilling on COG1 accumulation, the seedlings of *COG1-HA*-L1 were cold-treated then COG1-HA was detected at several timepoints. It was found that relative band densities of COG1-HA were 1.0, 3.2, 6.3, and 12.8 after chilling treatment for 0, 12, 24, 48 h, respectively (Fig. 4d). Therefore, *COG1* encodes a cold-induced LRR-RLP located in the plasma membrane and ER.

### Chilling treatment enhances the interaction between COG1 and OsSERL2

**Fig. 5:**
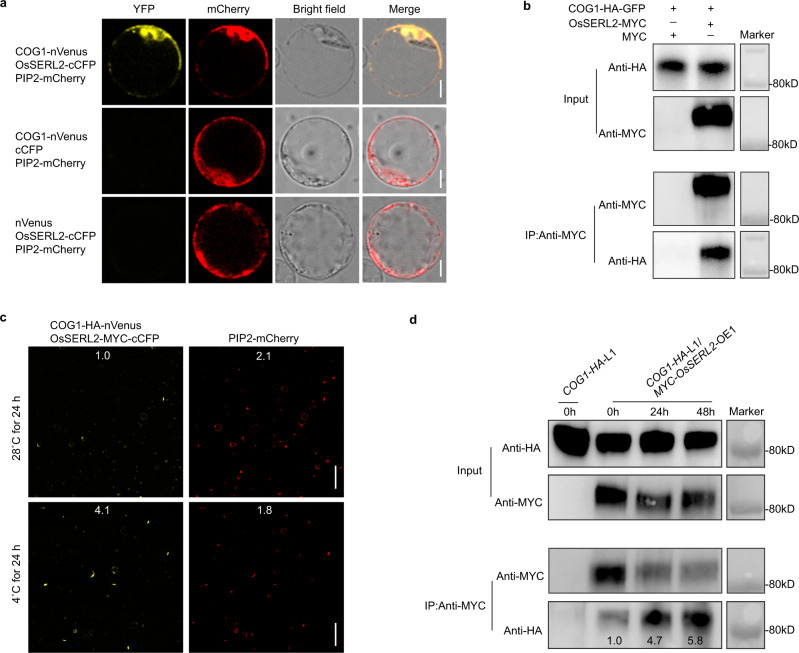
Chilling enhances the interaction between COG1 and OsSERL2. **a** Verification of the interaction between COG1 and OsSERL2 in rice protoplasts using BiFC assay. Empty cCFP and nVenus vectors were used as negative controls. Scale bar = 10 μm. **b** Co-immunoprecipitation (Co-IP) assay of COG1 and OsSERL2 in rice protoplasts. COG1-HA-GFP and MYC were co-expressed in rice protoplasts as negative controls. “+” or “–”denote the presence or absence, respectively, o f the protein in each sample. **c** Enhancement of the interaction between COG1 and OsSERL2 by cold in BiFC assay. The numbers at the top of each panels represent fluorescence intensity (mean gray value) as measured by Image J; values were normalized to the fluorescence in protoplasts co-expressing COG1-HA-nVenus and OsSERL2-MYC-cCFP at 28 °C w. Scale bar = 100 μm. **d** Enhancement of the interaction between COG1 and OsSERL2 by cold in Co-IP assay. The seedlings of *COG1-HA*-L1/*MYC-OsSERL2*-OE1 (see Methods) were treated at 4 °C for 0, 24, and 48 h and used for Co-IP. The relative intensity of the COG1-HA band was measured by Image J and the lane in *COG1-HA*-L1/*MYC-OsSERL2*-OE1 at 0 h was set as 1.0. Representative figures of **a**–**d** from three biological repeats. See also Supplementary Fig. 4. Source data are provided as a Source Data file.

The membrane localized LRR-RLPs can transmit cellular signals through interactions with SERK family members^22,23^. Based on the DNA sequences, rice genome is predicted to encode two SERKs and seven SERLs^28^ (http://rice.uga.edu/). The interactions between COG1 and these nine OsSERK/OsSERL family members were detected by yeast two-hybrid assays. The data showed that OsSERL2 had a stronger interaction with COG1 than OsSERL4, OsSERL5, OsSERK1 or OsSERK2 did (Supplementary Fig. 4a). These interactions were further confirmed in plant cells via bimolecular fluorescence complementation (BiFC) assays. COG1-nVenus was co-expressed in rice protoplasts with OsSERL2-cCFP, OsSERL4-cCFP, OsSERL5-cCFP, OsSERK1-cCFP, and OsSERK2-cCFP, respectively. Data showed only the protoplasts co-expressing COG1-nVenus with OsSERL2-cCFP produced a clear YFP fluorescence signal (Supplementary Fig. 4b and Fig. 5a). Furthermore, co-immunoprecipitation (Co-IP) assays showed that COG1 interacted specifically with OsSERL2 (Fig. 5b). The confocal observation found that COG1 co-localized with OsSERL2 in the plasma membrane (Supplementary Fig. 4c). Thus, both in vitro and in vivo data support that COG1 physically interacts with OsSERL2.

To explore the effect of temperature on the interaction between COG1 and OsSERL2, rice protoplasts co-expressing COG1-HA-nVenus and OsSERL2-MYC-cCFP were treated at 28 °C or 4 °C for 24 h, then the fluorescence densities were compared. The confocal observation showed that the fluorescence intensity in protoplasts co-expressing COG1-HA-nVenus and OsSERL2-MYC-cCFP was obviously higher at 4 °C (4.1) than at of 28 °C (1.0), but the protein abundance of COG1-HA-nVenus or OsSERL2-MYC-cCFP was constant after the cold treatment (Fig. 5c and Supplementary Fig. 4d). To confirm these results, *COG1-HA*-L1 and *COG1-HA*-L1/*MYC-OsSERL2*-OE1 seedlings were treated at 4 °C and Co-IP assays were performed with equivalent levels of COG1-HA in the total proteins. The immunoblotting showed that the relative density of COG1-HA band was increased to 4.7 and 5.8 after 24 and 48-hour treatment, respectively, compared with 0-hour treatment (1.0) (Fig. 5d). Collectively, these results suggested that cold stress strengthen the interaction between COG1 and OsSERL2.

### COG1 promotes the activation of OsSERL2 for enhancing cold tolerance

**Fig. 6:**
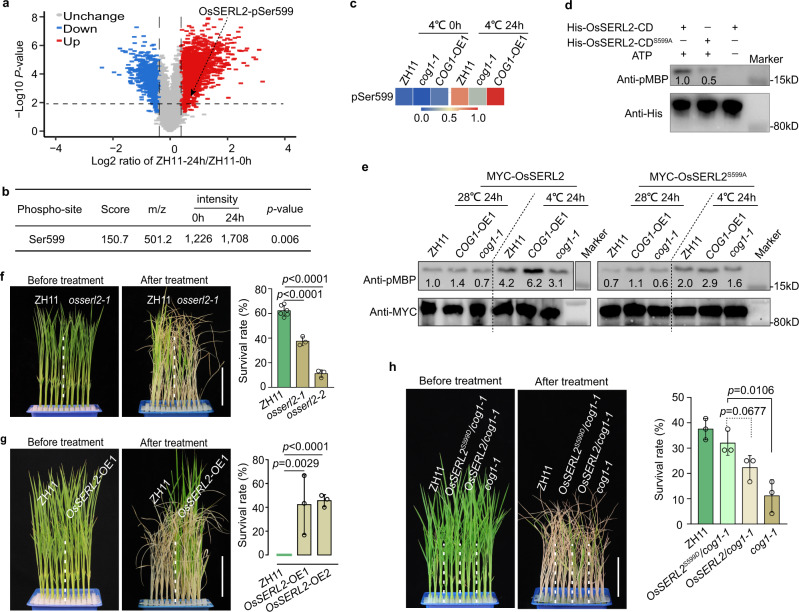
COG1 promotes the activation of OsSERL2 to enhance cold tolerance. **a** The volcano plot of TMT-labeled phosphorylation proteomics. Phosphorylation levels are shown for proteins extracted from ZH11 seedlings after treatment at 4 °C for 0 and 24 h. OsSERL2 phosphorylation at Ser599 (pSer599) was induced after 24 h of chilling. Data are shown as the means of three biological replicates (two-tailed Student’s *t*-test). **b** The phosphorylation intensity of OsSERL2 at Ser599 identified by TMT-labeled phosphorylation proteomics after treatment at 4 °C for 0, 24 h. Data are shown as the means of three biological replicates (two-tailed Student’s *t*-test). **c** Quantification of OsSERL2-pSer599 levels in ZH11, *cog1-1* and *COG1*-OE1 as identified with LC-MS/MS using the parallel reaction monitoring (PRM) method. The phospho-peptide fragment ions peak area values were normalized by min-max normalization. The detailed data were showed in Supplementary Data 4. **d** The influence of pSer599 on activation of OsSERL2 in vitro. Anti-phosphorylated myelin basic protein (pMBP) antibody was used to detect the kinase activity of cytoplasmic domain of OsSERL2 (His-OsSERL2-CD) and a version with a Ser to Ala mutation at site 599 (His-OsSERL2-CD^S599A^), and anti-His antibody was used to detect the amount of protein added. “+” or “–”denote the presence or absence, respectively, of the protein in each sample. Similar results were observed from three independent biological repeats. **e** Kinase activity of MYC-OsSERL2 and MYC-OsSERL2^S599A^ in ZH11, *cog1-1* and *COG1*-OE1 seedlings after cold stress. Anti-MYC antibody was used to detect MYC-OsSERL2 and MYC-OsSERL2^S599A^ in the immunoprecipitation product. Representative figures from three biological repeats. **f**, **g** The chilling tolerance of *osserl2* mutants (**f**) and *MYC*-*OsSERL2* overexpression lines (*OsSERL2-*OE1 and *OsSERL2-*OE2) (**g**) were treated at 4 °C for 70 h and 84 h. Bar = 6 cm. The survival rates were determined after a 4-week recovery period. Data are means ± SD (two-tailed Student’s *t*-test; *n* = 3 biological replicates). **h** The chilling tolerance of *35S::MYC-OsSERL2*^*S599D*^/*cog1-1* (*OsSERL2*^*S599D*^/*cog1-1*) and *35S::MYC-OsSERL2*/*cog1-1* (*OsSERL2*/*cog1-1*) seedlings were treated at 4 °C for 78 h. Bar = 6 cm. The survival rates were determined after a 4-week recovery period. Data are means ± SD (two-tailed Student’s *t*-test; *n* = 3 biological replicates). See also Supplementary Fig. 5. Source data are provided as a Source Data file.

To understand how COG1 response to cold stress by interacting with OsSERL2, the Tandem Mass Tag (TMT)-labeled high-throughput phosphorylation proteomics approach was used. The mass spectrometry (MS) results showed that the phosphorylation of OsSERL2 at Ser599 was induced after 24-h chilling treatment in ZH11 (Fig. 6a, b and Supplementary Fig. 5a). To exclude that the induction of phosphorylation of OsSERL2 resulting from the protein accumulation, *OsSERL2::OsSERL2-MYC* was expressed in rice protoplasts. The protein level of OsSERL2 did not significantly change after the treatment (Supplementary Fig. 5b). To test the effect of COG1 on cold-induced phosphorylation of OsSERL2, phospho-Ser599 (pSer599) levels of OsSERL2 were detected in ZH11, *cog1-1* and *COG1*-OE1 using the Parallel Reaction Monitoring (PRM) method^46^. The results showed that pSer599 levels were lower in *cog1-1* but higher in *COG1*-OE1 than in ZH11 after chilling treatment (Fig. 6c, Supplementary Fig. 5c and Supplementary Data 4). The kinase activity of OsSERL2 may have been affected by pSer599, which is in the C-terminal tail of OsSERL2 (Supplementary Fig. 5d)^47,48^. Kinase activity were therefore detected in the cytoplasmic domain of OsSERL2 (OsSERL2-CD, residues 290–641) and a mutated form of OsSERL2-CD, in which Ser599 was substituted with Ala599 (OsSERL2-CD^S599A^), using the general substrate myelin basic protein (MBP). The phospho-MBP levels decreased from 1.0 to 0.5 in OsSERL2-CD^S599A^ compared to OsSERL2-CD (Fig. 6d), implying that the phosphoralation on Ser599 is essential for the activation of OsSERL2.

Based on the results showing that pSer599 level was affected by COG1 (Fig. 6c) and appeared essential for kinase activity of OsSERL2 (Fig. 6d), it can be speculated that COG1 may mediated OsSERL2 kinase activity by regulating pSer599 level. To determine this speculation, the activity of MYC-OsSERL2 was detected in the protoplasts of ZH11, *cog1-1* and *COG1*-OE1. The immunoblotting results showed that the cold-induced kinase activity of MYC-OsSERL2 was lower in *cog1-1* but higher in *COG1*-OE1 than in ZH11 after chilling treatment (Fig. 6e). When Ser599 was mutated to Ala, the kinase activity of MYC-OsSERL2^S599A^ was weakened in vivo (Fig. 6e). These results suggested that COG1 promotes the cold-induced activation of OsSERL2 through enhancing pSer599 level.

In order to test the function of *OsSERL2* in chilling tolerance, loss-of-function mutants (*osserl2-1* and *osserl2-2*) and *MYC-OsSERL2* overexpression lines (*OsSERL2-*OE1 and *OsSERL2-*OE2) were generated in the ZH11 background (Supplementary Fig. 5e, f). After the seedlings were treated at 4 °C for 70 h, the survival rates of *osserl2-1* and *osserl2-2* (38% and 11%, respectively) were significantly lower than that of ZH11 (63%) (Fig. 6f). In contrast, the survival rates of the overexpression lines were higher than that of ZH11 after treatment at 4 °C for 84 h (Fig. 6g). To demonstrate the genetic function of pSer599 in OsSERL2, the *35S::MYC-OsSERL2* and *35S::MYC-OsSERL2*^*S599D*^ (Ser599 was substituted with Asp599 to mimick phospho-Ser) transgenic plants in *cog1-1* background were generated. After the seedlings were treated at 4 °C for 78 h, the survival rates of *35S::MYC-OsSERL2*^*S599D*^/*cog1-1* (32%) was significantly higher than that of *cog1-1* (11%), or that of *35S::MYC-OsSERL2*/*cog1-1* (22%) (Fig. 6h and Supplementary Fig. 5g). These results suggested that COG1 promotes the activation of OsSERL2 relying on pSer599 to enhance cold tolerance.

### The COG1-OsSERL2 complex causes the activation of OsMAPK3 to transmit cold signal

**Fig. 7:**
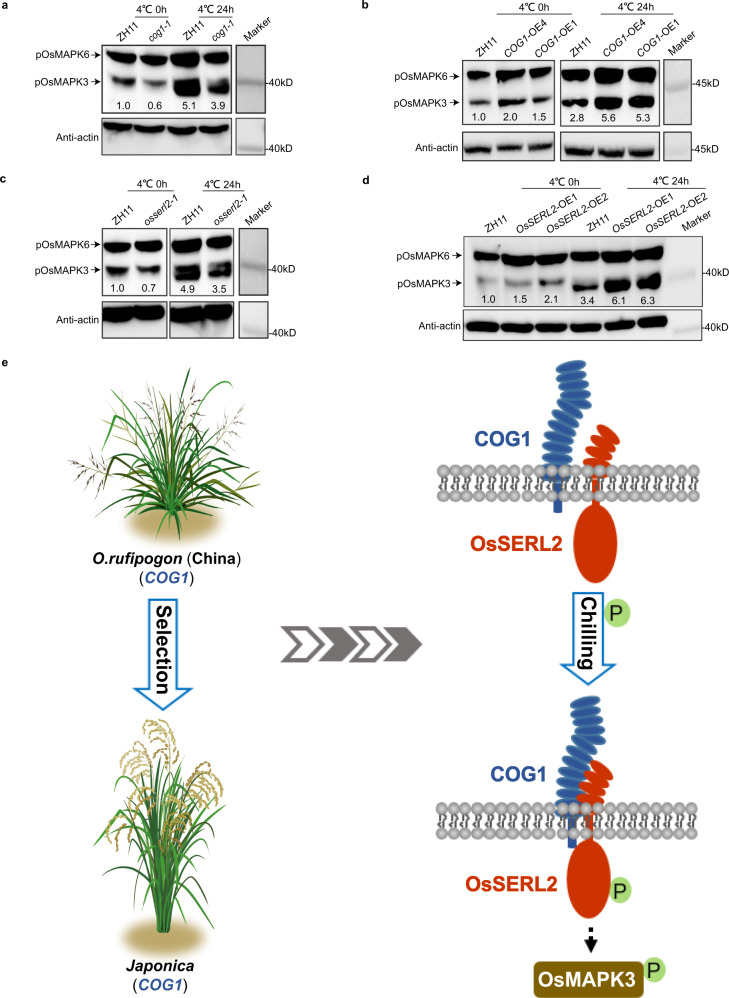
The COG1-OsSERL2 complex causes the activation of OsMAPK3 to transmit cold signal. **a**, **b**, **c** and **d** The phospho-Thr194 and phospho-Tyr196 levels of OsMAPK3 (pOsMAPK3) in *cog1-1* (**a**), in *COG1* overexpression lines (*COG1*-OE1 and *COG1*-OE4) (**b**), in *osserl2-1* (**c**), and in *MYC*-*OsSERL2* overexpression lines (*OsSERL2*-OE1 and OE2) (**d**). Total protein extracts were used for immunoblotting with anti-pTEpY and anti-actin antibodies. The relative band intensities were measured with Image J. The ratio of the band intensities of pOsMAPK3 to actin in ZH11 after 4 °C treatment for 0 h was set to 1.0. Representative figures of a–d from three biological repeats. **e** Working model of COG1. *COG1* might originated from Chinese populations of *O. rufipogon* and been under selection in *japonica* rice during domestication. The receptor like protein COG1 interacts with OsSERL2 and this interaction is enhanced by chilling. The interaction promotes activation to OsSERL2 for sensing cold signals. The COG1-OsSERL2 complex causes the activation of OsMAPK3 to transmit cold signal from the membrane to the cytoplasm, enhancing cold tolerance in rice. See also Supplementary Fig. 6. Source data are provided as a Source Data file.

As known, MAPK cascades are key signaling modules downstream of RLKs/RLPs receptor complexes^29^. OsMAPK3 is a critical protein in the rice MAPK cascade that is specifically induced by chilling^35,49^. Our phosphorylation proteomics data also showed that the phosphorylation levels of Thr194 and Tyr196 in OsMAPK3 were induced by chilling (Supplementary Fig. 6a–c). To confirm whether COG1-OsSERL2 could activate the OsMAPK3 cascade in chilling signaling, the phospho-Thr194 and phospho-Tyr196 levels of OsMAPK3 (pOsMAPK3) were detected in *COG1* and *OsSERL2* transgenic plants using anti-pTEpY antibody^49,50^. Compared with wild-type ZH11, the pOsMAPK3 levels were decreased in *cog1-1* (Fig. 7a) but increased in *COG1* overexpression lines after chilling treatment (Fig. 7b). Furthermore, the pOsMAPK3 levels were also decreased in *osserl2-1* but increased in *MYC-OsSERL2* overexpression lines compared to ZH11 after chilling treatment (Fig. 7c and d). Consistent with the antibody assay results, the PRM assay also showed that the pOsMAPK3 levels were decreased in *cog1-1* but increased in *COG1*-OE1 compared to ZH11 after chilling treatment (Supplementary Fig. 6d, e). Therefore, the COG1-OsSERL2 complex caused the activation of OsMAPK3 to transmit cold signal from the membrane to the cytoplasm (Fig. 7e).

## Discussion

The trait of chilling tolerance is genetically controlled by multiple genetic loci. As known, the chilling tolerance gene *COLD1*, which is found in *japonica* accessions with a specific SNP2, originated from the Chinese population of *O. rufipogon*^2^. Alternatively, phylogenetic and population genetic analyses here suggested that *COG1* in *japonica* accessions with haplogroup1 originated from Chinese *O. rufipogon* populations and was fixed in *japonica* due to strong human selection during domestication (Fig. 3). *COG1* is a novel major gene of *qCS11-jap*, which confers chilling tolerance in *japonica* rice but make sensitive to the tolerance in *indica* background (Fig. 1 and Fig. 2). Natural populations showed that the functional *COG1* was fixed only in chilling-tolerant subspecies (*japonica* rice) and negatively selected in *indica* rice (Supplementary Data 3 and Fig. 3). Our findings are consistent with archeological and genetic evidence of *japonica* domestication, *japonica* rice originated in the Yangtze Valley of China ~9000 years ago then underwent a low-temperature bottleneck, forming both temperate and tropical *japonica* varieties during a global cooling event ~4200 years ago^7,44^. Importantly, our work provides another piece of evidence that temperature is a major factor influencing rice domestication^2,3,44^. The fixation and extension of favored alleles that enhance chilling tolerance to maintain growth of *japonica* rice in regions with relatively low yearly temperatures.

It is extensive application potential in rice breeding that the epistasis traits of QTL genes depend on the various genetic background^38–40,51^, such as *COG1*. The opposite phenotype of *COG1* transgenic rice in *japonica* and *indica* background may be explained with a model of genetic incompatibilities: invasion of *COG1* disturbs endogenous OsSERL2^*indica*^ function, causing MAPK cascade down-regulation in *indica* background (Fig. 1, Fig. 2 and Supplementary Fig. 7)^34,52–55^. Specifically, OsSERL2^*indica*^ may have evolved a parallel activation pathway aided by intrinsic RLP/RLK in the absence of *COG1*; in *indica* genome, *OsSERL2* has variations to encode the extracellular domain and C-terminal in genome (https://snp-seek.irri.org/). Interaction of exogenous COG1 with OsSERL2^*indica*^ may disturb the critical interaction between OsSERL2^*indica*^ and intrinsic RLP/RLK, decreasing OsMAPK3 activation (Supplementary Fig. 7)^54,55^. Thus, exogenous COG1 from *japonica* rice would disrupt the intrinsic OsSERL2^*indica*^-mediated chilling tolerance signaling pathway in *indica* background. The epistasis effect between COG1 and a hypothetical factor in *indica* varieties may therefore inhibit improvement of traits such as cold tolerance. The identified negative effects can be improved with new technology, such as the gene-editing, to break the linkage drag in molecular breeding. This would promote maintenance of cold-tolerance or other key traits in breeding populations when introgression genes between *japonica* and *indica* varieties are used in rice production^51,56^.

It is the first reported that an LRR-RLP (COG1) with a co-receptor (OsSERL2) senses cold for the tolerance. Evidence from the membrane localization, protein interaction and kinase activity dependent on cold stimulating (Figs. 4–6), as well, downstream responses may suggest the COG1-OsSERL2 complex senses cold to transmit the signaling for tolerance (Fig. 7). RLPs typically depend on co-receptor kinases to perceive and respond to cold stress‐induced ligands for activating intracellular signaling; this is analogous to hetero‐dimerization of RLKs^12,13,22,23^. However, no ligand for perceiving the cold signal has been reported in plant cells. Changes in the conformation of proteins such as OsCRT3/OsCIPK7 are considered to be another potential mechanism for cold perception in plants^57–59^. The membrane-localized COG1-OsSERL2 complex senses cold through conformational changes, activating the kinase to transduce a signal to the MAPK cascade, ultimately inducing a cold-tolerance response. Conformational changes of the complex to reinforce heterodimerization may be involved in the dynamic cold-sensing process; this could be confirmed in the further through several new approaches, such as, Fourier-transform infrared (FTIR) spectroscopy, Cryo-EM assay^59,60^.

As known, the sensing of signals in the apoplast by RLK-RLK complexes typically leads to phosphorylation and activation of intracellular domain by transphosphorylation^23,29^. With respect to the RLP modulation of RLK in signal sensing, our evidence showed that COG1 may act as an adapter based on its biochemical characteristics (Fig. 4 and Fig. 5). Adapter proteins can affect the phosphorylation status of interacting proteins^61,62^. Our findings indicate that COG1 affect phosphorylation levels of OsSERL2 at Ser599, regulating kinase activity of OsSERL2 in cold signal transduction (Fig. 6). This study uncovered a potential role of the COG1-OsSERL2 complex in perceiving and transmitting the cold signal in rice.

Signals including cold, sensed by the RLP-RLK receptor complexes in the membrane are transduced through the MAPK cascade to the cytoplasm (Fig. 7)^29^. For example, a cascade involving YDA-MKK4/MKK5-MPK3/MPK6 participates in transmitting stomatal development signal sensed by the AtRLP17-AtSERK3 receptor complex^12^. The OsRLCK185-OsMAPKKKε-OsMKK4-OsMAPK3 cascade transmits chitin-related signal after it is sensed by the OsCEBiP-OsCERK1 complex^63^. The activated OsMAPK3 directly phosphorylates the transcription factors OsbHLH002^35^ or OsWRKY53^64^, altering the expression of cold-regulated genes. Our phosphoproteomics date also showed an increase of phospho-OsWRKY53 (Supplementary Data 6). Here, our data suggest that the activation of OsMAPK3 relied on the COG1-OsSERL2 complex in cold signaling pathway (Fig. 7). Overall, our results shed light on a panorama of how a major QTL gene *COG1* modulates chilling tolerance in rice.

In summary, *COG1* is a major gene of the QTL to positively modulate chilling tolerance in *japonica* rice. *COG1* originated from Chinese populations of *O. rufipogon* and was selected during *japonica* rice domestication. The LRR-RLP protein COG1 forms a complex with OsSERL2 to promote its activation for sensing cold signal. The COG1-OsSERL2 complex senses and transmits cold signal from the apoplast to the cytoplasm to activate the MAPK cascade, triggering defenses against chilling stress in *japonica* rice (Fig. 7e). This novel cold-sensing module is potentially used in molecular design for rice breeding to improve the traits.

## Methods

### Genetic population and plant materials

The chromosome segment substitution line (CSSL) population was generated by crossing and back-crossing using the chilling-sensitive *indica* variety 93-11 as the recurrent parent and the chilling-tolerant *japonica* variety Nipponbare as the donor parent^37^. The chilling-sensitive line CSSL-28 was selected after evaluating the chilling tolerance of CSSLs at seedling stage. For QTL genetic assay, the F_2:3_ population consisting 1800 individuals was generated by crossing the CSSL-28 with 93-11, self-crossing the progeny. A homozygous recombinant line (R2-7-4) was obtained from F_2:3_ the population through molecular marker assisted selection. The primers used are shown in Supplementary Data 5.

To generate the mutants of *COG1* and *OsSERL2* through CRISPR/Cas9 genome editing system, the target site sequences 5'-GAACAACAACAGTGAGAAGC-3' (*COG1*) and 5'-GCTCCCCGGATTTCCTCGT-3' (*OsSERL2*) were separately cloned into a CRISPR/Cas9 vector linearized with *BsaI* (Biogle Cat# BGK03) following manufacturer’s instruction. The full coding sequences of *COG1* was amplified from cDNA of ZH11 using the primers pUN1301*-COG1*-F/R, then cloned into pUN1301, a binary vector that carries the maize ubiquitin gene promoter; the vector had been linearized with *Bam*HI and *Kpn*I (Supplementary Data 5). The construct was transformed in ZH11 and 93-11, respectively, to generate *COG1* overexpression lines in both backgrounds. The full coding sequences of *OsSERL2* was amplified from cDNA of ZH11 using the primers pUN1301-*MYC-OsSERL2*-F2/R2 and 6×*MYC* fragment was amplified from pBI221-OsbHLH002-MYC construct^49^ using the primers pUN1301-*MYC-OsSERL2*-F1/R1. The two fragments were assembed into pUN1301 (which had been linearized with *Bam*HI and *Kpn*I) to generate the pUN1301-*MYC-OsSERL2* construct (Supplementary Data 5). The construct was transformed in ZH11 to generate *MYC*-*OsSERL2* overexpression line. Genomic DNA of *COG1* comprising promoter fragment (1751-bp) and full coding sequences were amplified from ZH11 using the primers *COG1::COG1*-F/R. The product was cloned into the pTFLGHAU vector, which had been linearized with *ECoRI* and *BamHI*, to obtain the *COG1::COG1-HA* construct (Supplementary Data 5). The construct was transformed in ZH11 and 93-11, respectively, to generate *COG1::COG1-HA* transgenic lines in both background. *Agrobacterium tumefaciens* strain EHA105-mediated transformation was used to introduce the constructs into rice^2^. *COG1-HA*-L1/*MYC-OsSERL2*-OE1 line was obtained by crossing *COG1::COG1-HA* transgenic line 1 (*COG1-HA*-L1 in the ZH11 background) with the *MYC*-*OsSERL2* overexpression line (*MYC-OsSERL2*-OE1). *MYC-OsSERL2* and its mutated form with 1795T > G, 1796C > A (Ser599 were mutated to Asp599, *MYC-OsSERL2*^*S599D*^) were each amplified from the pUN1301-*MYC-OsSERL2* construct (Supplementary Data 5). The two products were separately cloned into WMV049 (wimibio), which had been linearied with *Kpn*I and *Bam*HI. The resulting constructs were transformed in the *cog1-1* background to generate the *35S::MYC-OsSERL2/cog1-1* and *35S::MYC-OsSERL2* ^*S599D*^*/cog1-1* transgenic lines, respectively.

### Chilling treatment

Chilling treatments were performed as described in our previous report with slight modifications^65^. The undehulled seeds were surface-sterilized with 0.6% sodium hypochlorite solution for 15 min, then rinsed three times with sterile distilled water. Sterilized rice seeds were immersed in water for 2 days at 30 °C. After germination, seeds were sown in an incubator with Kimura B nutrient solution and the incubators were placed in a greenhouse with a light intensity of 120 μmol·m^–2^·s^–1^ at 28/25 °C (day/night) cycles and a short-day photoperiod (10-h day/14-h night). After 2 weeks, seedlings were moved to a water bath, and ~3 cm of the aerial tissue was immersed in water for chilling treatment. The water bath was maintained at 3.8–4.1 °C and in a room at 20 ± 2 °C with the same photoperiod as that in the greenhouse. Each replicate contained >24 seedlings with consistent growth. After cold treatment, seedlings were returned to the greenhouse for recovery. The survival rate (percentage of living seedlings out of all tested plants) was determined after a 4-week recovery period. Each treatment was performed independently at least three times. Significan differences between treatment groups were assessed with Student’s *t*-test.

### Variations identification, genetic diversity, and phylogenetic analysis

The pan-genome dataset (containing 67 representative rice accessions) was downloaded from RicePanGenome website (http://db.ncgr.ac.cn/RicePanGenome/)^41^. Variations in the coding region of *COG1* were called using genomes from the entire dataset. *COG1* sequences from all 67 representative rice accessions were aligned to construct a phylogenetic tree using the neighbor-joining method in MEGA 5.0^66^. Nucleotide diversity analyses of *COG1* were performed for each rice subspecies group using DnaSP 5.1^67^. Resequencing data from 3000 Rice Genomes project were downloaded from the Rice SNP-Seek Database (https://snp-seek.irri.org/)^68^. The resequencing data of the 1176 landraces in 3000 Rice Genomes Project were selected as in previous studies^8,44^. The likelihood-based selective sweep detection was calculated by ‘SweeD’ with a 1 kb grid size^69^.

### Protein extraction and immunoblotting

Total proteins were extracted from 14-day-old rice seedlings and rice protoplasts using a buffer consisting of 50 mM Tris-HCl (pH 7.5), 150 mM NaCl, 10% glycerol, 5 mM dithiothreitol (DTT), 1% (v/v) protease inhibitor cocktail (P9599, Sigma), 1% (v/v) IGEPAL CA-630, 1 mM phenylmethanesulfonyl fluoride PMSF (PMSF), 1 mM Na_2_MoO_4_·2H_2_O, 1 mM NaF, 1.5 mM Na_3_VO_4_, and 1% (v/v) protein phosphatase inhibitor cocktails 2 and 3 (Sigma, Cat# P5726 and P0044).

For immunoblotting, the proteins were separated by SDS-PAGE (Genscript, Cat# M00661) in 1× MOPS buffer (Genscript, Cat# M00138), then transferred to a PVDF membrane (Millipore Cat# IPVH00010) via electro-transfer at 120 mA for 90 min. The membrane was incubated in blocking buffer (5% w/v nonfat dry milk, 1×TBS, and 0.1% Tween® 20) for 3 h at room temperature, incubated first with the primary antibody for 8 h, followed by the secondary antibody for 1 h (anti-rabbit IgG, Cat# 7074, anti-mouse IgG, Cat# 7076, Cell Signaling Technology, dilution, 1:5000).

To analyze the effects of chilling on COG1 accumulation, 14-day-old seedlings of *COG1-HA*-L1 were treated at 4 °C for 0, 12, 24, and 48 h. The total proteins were extracted for immunoblotting using anti-HA antibody (Sigma, Cat# H6908, dilution, 1:10,000). The same method was used to detect the COG1-HA with anti-HA antibody, MYC-OsSERL2 with anti-MYC antibody (CWBIO, Cat# CW0299M, dilution, 1:3000) and Rubisco with anti- Rubisco antibody (Agrisera, Cat# AS10700, dilution, 1:5000) from the corresponding transgenic rice, respectively.

To analyze subcellular localization of COG1, proteins were extracted from cell fractions of *COG1-HA*-L1 14-day-old rice seedlings using the Minute™ Plasma Membrane Protein Isolation and Cell Fractionation Kit (invent, Cat# SM-005-P). The proteins were detected by SDS-PAGE using anti-HA, anti-H^+^-ATPase (Agrisera, Cat# AS07260, dilution, 1:5000), anti-BiP (YOUKE, Cat# YKZP054, dilution, 1:2000), anti-actin (Huaxingbio, Cat# HX1843, dilution, 1:5000), and anti-histone 3 (Earthox Cat# E022020, dilution, 1:10,000) antibodies, respectively.

### Yeast two-hybrid (Y2H) assays

Full-length coding sequences of rice SERK/SERL family members and *COG1* were amplified from cDNA of ZH11 using the primers shown in Supplementary Data 5. Amplicons were cloned into the DUALmembrane system vectors pBT3-STE and pPR3-N (Dualsystems Biotech, Cat# P01201-P01229) after the vectors were linearized with *Sfi*I, respectively. The pBT3-STE*-OsSERK/SERLs* and pPR3-N*-COG1* vectors were transformed into yeast strains NMY51, following the manufacturer’s instructions of DUALmembrane starter kits.

### Co-IP analysis

To verify the interaction between COG1 and OsSERL2 in vivo, the coding sequences of *COG1* and a 3×*HA* fragment were amplified from *COG1::COG1-HA* construct using the primers pBI221-*COG1-HA-GFP*-F1/R1 and pBI221-*COG1-HA-GFP*-F2/R2, respectively. These fragments were assembed into the pBI221 vector, which had been linearized with *Xho*I and *Kpn*I (Supplementary Data 5). The coding sequences of *OsSERL2* and a 6×*MYC* fragment were amplified from pUN1301-*MYC-OsSERL2* construct using the primers pBI221-*OsSERL2-MYC*-F1/R1 and pBI221-*OsSERL2-MYC*-F2/R2, respectively. These fragments were inserted into the pBI221 vector after the sequence encoding the GFP-tag was removed with *KpnI* and *SacI* (Supplementary Data 5). The resulting plasmids were then transfected into rice protoplasts to perform Co-IP (co-immunoprecipitation) assays as previously described^49^. The Co-IP products were immunoprecipitated from the total protein mixtures using Anti-c-Myc Magnetic Beads (Pierce, Cat# 88843), then separated via 8% SDS–PAGE and detected with anti-MYC and anti-HA antibodies. To determine whether cold affected the interaction between COG1 and OsSERL2, the seedlings of *COG1-HA*-L1/*MYC-OsSERL2-*OE1 were treated at 4 °C for 0, 24, and 48 h and the *COG1-HA*-L1 were treated at 4 °C for 0 h. Total proteins were extracted from the seedlings and adjusted to contain equivalent levels of COG1-HA prior to immunoprecipitation assays.

### Subcellular localization and BiFC assays

To analyze subcellular localization of COG1 in rice protoplasts, the coding sequences of *COG1* amplified from cDNA of ZH11 using the primers pBI221*-COG1-*F/R, then cloned into pBI221 (which had been linearized with *Xho*I and *Kpn*I). To determine whether COG1 co-localized with OsSERL2 in rice protoplasts, the coding sequences of *COG1* was amplified using the primers pBI221*-COG1-mCherry-*F1/R1 and the *mCherry* fragment was amplified from the PIP2-mCherry construct^2^ using the primers pBI221*-COG1-mCherry-*F2/R2. The two fragments were then assembed in the pBI221 vector *Kpn*I and *Sac*I. The coding sequences of *OsSERL2* was amplified from cDNA of ZH11 using the primers pBI221-OsSERL2-GFP-F/R, then cloned into pBI221 (which had been linearized with *XhoI* and *KpnI*) (Supplementary Data 5). For BiFC assays in rice protoplasts, the coding sequences of *COG1*, *COG1-HA*, *OsSERL2*, and *OsSERL2-MYC* were amplified from the pBI221-*COG1-HA-GFP* and pBI221-*OsSERL2-MYC* constructions using the primers shown in Supplementary Data 5, then separately inserted into the pSAT1-cCFP-C and pSAT1-nVenus-C vectors (which had been linearized with *Hin*dIII and *Xba*I). The coding sequences of *OsSERL4*, *OsSERL5*, *OsSERK1*, *OsSERK2* were amplified from cDNA of ZH11, then cloned into pSAT1-nVenus-C using the same method (Supplementary Data 5). Protoplasts were transfected as described above. The transfected protoplasts were observed using a fluorescence microscope (Leica TCS SP5). Images were analyzed with Image LAS-AF software.

### In vivo phosphorylation assays

The phospho-OsMAPK3 levels in Zhonghua 11 (ZH11), *cog1-1*, *COG1* overexpression lines, *osserl2-1* and *MYC-OsSERL2* overexpression lines were detected by anti-pTEpY antibody using the method as described in our previous report^49^.

### In vitro and in vivo protein kinase activity detection

For in vitro protein kinase activity detection, the coding sequences of *OsSERL2* containing 870–1923 (cytoplasmic domain, OsSERL2-CD) and its mutated form containing a 1795T > G mutation (Ser599 were mutated to Ala599, OsSERL2-CD^S599A^) were amplified from the pUN1301-*MYC-OsSERL2* construct, respectively. Each amplicon was separately cloned into pColdTF vector which contained a His tag after linearization with *Sac*I and *Xba*I (Supplementary Data 5). His-OsSERL2-CD and His-OsSERL2-CD^S599A^ were purified by a prokaryotic expression system. A kinase assay was performed in the presence of 50 mM ATP and 30 μL of kinase buffer (25 mM Tris-HCl pH 7.5, 5 mM beta-glycerophosphate, 2 mM dithiothreitol, 0.1 mM Na_3_VO_4_, and 10 mM MgCl_2_.) containing the substrate Myelin Basic Protein (MBP) and either purified His-OsSERL2-CD or His-OsSERL2-CD^S599A^. Then the mixtures were incubated at 30 °C for 30 min. The reactions were stopped by adding SDS-loading buffer. The reaction mixtures were separated by 12% SDS-PAGE for immunoblotting analysis using anti-His (Earthox, Cat# E022020, dilution, 1:5000) and anti-pMBP (Sigma, Cat# 05-429, dilution, 1:5000) antibodies.

For in vivo protein kinase activity detection, the coding sequences of *MYC-OsSERL2* and its mutated form containing a 1795T > G mutation (*MYC-OsSERL2* ^*S599A*^) were amplified from the pUN1301-*MYC-OsSERL2* construct and cloned into pBI221 with *Kpn*I and *Sac*I (Supplementary Data 5). The resulting plasmids were transfected into protoplasts of ZH11, *cog1-1* and *COG1*-OE1. The transformed protoplasts were cultured at room temperature for 16 h then at 28 °C or 4 °C for 24 h. After that, total protein was extracted from the protoplasts and immunoprecipitated by Anti-c-Myc Magnetic Beads. The IP products were used for kinase activity detection and performed as described above.

### Quantitative RT-PCR

RNA was extracted from whole seedlings using a plant RNA kit (ZOMANBIO, Cat# ZP405K) following the manufacturer’s instructions. First-strand complementary DNA was synthesized from 2 µg total RNA using a cDNA synthesis kit (YEASEN, Cat# 10911). Quantitative real-time PCR assays were performed using the SYBR Green Realtime PCR Master Mix (TOYOBO, Cat# QPK-201). All primers used for qRT-PCR are given in Supplementary Data 5.

### Quantitative phosphoproteomics detection

#### TMT-labeled quantitative phosphorylation detection

The 14-day-old rice seedlings of ZH11 were treated at 4 °C for 0, 24 h, then proteins were extracted following previously described^70^. The protein concentration was determined by a BCA kit (Beyotime). Proteins were digested using trypsin (Promega) following the manufacturer’s instruction. For TMT labeling, trypsin-hydrolyzed peptides were desalted with strata XC18 (phenomenex) and freeze-dried under a vacuum. The peptides were dissolved in 0.5 M TEAB and labeled following the manufacturer’s instructions of the TMT-6plex kit (Thermo Fisher, Cat#90068). Phosphopeptide enrichment was performed using a TiO_2_ affinity technique (PTM BIO) following the manufacturer’s instruction. The LC-MS/MS analysis were performed as described in our previous report using EASY-nLC 1000 UPLC system and Q ExactiveTM Plus (Thermo Fisher)^70^. The resulting MS/MS data were processed using the MaxQuant search engine (v.1.6.15.0). Tandem mass spectra were searched against the *Oryza_sativa_subsp._japonica*_39947_PR_20200603.fasta database concatenated with a reverse decoy database. Trypsin/P was specified as the cleavage enzyme and up to two missing cleavages were allowed. The mass tolerance for precursor ions was 20 ppm in the first search and 4.5 ppm in the main search, and the mass tolerance for fragment ions was 0.02 Da. TMT-6plex quantification was performed. The false discovery rate was adjusted to <1% and the minimum score threshold for peptides was >40. For statistical analysis, the mean intensities at 24 h were divided by the mean intensities at 0 h to obtain the phosphorylation intensity ratios of ZH11-24h vs ZH11-0h. Significant differences were assessed with Student’s *t*-test.

To analyze the effects of chilling on OsSERL2 accumulation, *OsSERL2* promoter fragment (1710-bp) and the *OsSERL2-MYC* fragment were amplified from genomic DNA of ZH11 and the pBI221-*OsSERL2-MYC* construct, respectively. The two fragments were assembed into the pTYLUC vector after removing the sequence encoding the Luciferase-tag with *Sac*I and *Bam*HI, resulting in the *OsSERL2::OsSERL2-MYC* construct (Supplementary Data 5). The constructed plasmid was transfected into ZH11 protoplasts, which were treated as described above. After that, total proteins were extracted from the protoplasts and immunoblotting by Anti-MYC antibody.

#### Parallel reaction monitoring (PRM) quantitative phosphorylation detection

For PRM, the standard phosphopeptide: “ASGHSTAAADSLSHpSHR” for OsSERL2 and “SDMMpTEpYVVTR” for OsMAPK3 were synthesized. The synthesized standard phospho-peptides were loaded on by gradient and searched by maxquant. Then the retention time (the time elapsed from the start of the sample injected in chromatographic column until the appearance of the apex of chromategram of that sample), m/z values, N-terminal fragments (b ions) and C-terminal fragments (y ions) spectra of the phosphopeptide in the mass spectrum were obtained. These b, y ion spectra were used as the background database.

Total proteins were extracted from 14-day-old rice seedlings of ZH11, *cog1-1* and *COG1*-OE1 after treatment at 4 °C for 0, 24 h. The protein trypsin digestion and phosphopeptide enrichment were manipulated as described above. The phosphopeptide of each sample was analysed under PRM using the same machine and parameters with LC-MS/MS analysis^46^.

### Reporting summary

Further information on research design is available in the Nature Portfolio Reporting Summary linked to this article.

### Supplementary information

Supplementary Information

Description of Additional Supplementary Files

Supplementary Data 1

Supplementary Data 2

Supplementary Data 3

Supplementary Data 4

Supplementary Data 5

Supplementary Data 6

Reporting Summary

### Source data

Source Data

## Data Availability

The mass spectrometry proteomics data have been deposited to the ProteomeXchange Consortium via the PRIDE partner repository with the dataset identifier PXD042294. Data supporting the findings of this work are provided in the paper and its Supplementary Information file. Source data are provided with this paper.

## References

[1] Fairhurst T, Dobermann A (2002). Rice in the global food supply. Better Corps Int.

[2] Ma Y (2015). *COLD1* confers chilling tolerance in rice. Cell.

[3] Mao D (2019). Natural variation in the *HAN1* gene confers chilling tolerance in rice and allowed adaptation to a temperate climate. Proc. Natl. Acad. Sci. USA..

[4] Zhang Z (2017). Natural variation in *CTB4a* enhances rice adaptation to cold habitats. Nat. Commun..

[5] Ding Y, Yang S (2022). Surviving and thriving: how plants perceive and respond to temperature stress. Dev. Cell.

[6] Zhang J, Li XM, Lin HX, Chong K (2019). Crop improvement through temperature resilience. Annu. Rev. Plant Biol..

[7] Huang X (2012). A map of rice genome variation reveals the origin of cultivated rice. Nature.

[8] Wang W (2018). Genomic variation in 3,010 diverse accessions of Asian cultivated rice. Nature.

[9] Kim SI, Andaya VC, Tai TH (2011). Cold sensitivity in rice (*Oryza sativa* L.) is strongly correlated with a naturally occurring I99V mutation in the multifunctional glutathione transferase isoenzyme GSTZ2. Biochem. J.

[10] Lu G (2014). Rice LTG1 is involved in adaptive growth and fitness under low ambient temperature. Plant J.

[11] Wang G (2008). A genome-wide functional investigation into the roles of receptor-like proteins in Arabidopsis. Plant Physiol.

[12] Meng X (2015). Differential function of Arabidopsis SERK family receptor-like kinases in stomatal patterning. Curr. Biol..

[13] Jones DS, John A, VanDerMolen KR, Nimchuk ZL (2021). CLAVATA signaling ensures reproductive development in plants across thermal environments. Curr. Biol..

[14] Wolf S, van der Does D, Ladwig F, Sticht C, Kolbeck A (2014). A receptor-like protein mediates the response to pectin modification by activating brassinosteroid signaling. Proc. Natl. Acad. Sci. USA..

[15] Albert I (2015). An RLP23-SOBIR1-BAK1 complex mediates NLP-triggered immunity. Nat. Plants.

[16] Jehle AK (2013). The receptor-like protein ReMAX of Arabidopsis detects the microbe-associated molecular pattern eMax from Xanthomonas. Plant Cell.

[17] Zhang Y (2010). Arabidopsis *snc2-1D* activates receptor-like protein-mediated immunity transduced through *WRKY70*. Plant Cell.

[18] Zhang W (2013). Arabidopsis RECEPTOR-LIKE PROTEIN 30 and receptor-like kinase SUPPRESSOR OF BIR1-1/EVERSHED mediate innate immunity to necrotrophic fungi. Plant Cell.

[19] Zhang L (2014). Fungal endopolygalacturonases are recognized as microbe-associated molecular patterns by the Arabidopsis receptor-like protein RESPONSIVENESS TO BOTRYTIS POLYGALACTURONASES. Plant Physiol.

[20] Zhang H (2021). A rice LRR receptor-like protein associates with its adaptor kinase OsSOBIR1 to mediate plant immunity against viral infection. Plant Biotechnol. J..

[21] Park HC, Kim DW, Park J, Baek D, Yun DJ (2021). AtLRRop2, an leucine-rich repeat-only protein, mediates cold stress response in *Arabidopsis thaliana*. Plant Biotechnol. Rep..

[22] Liebrand, T. W. H., van den Burg, H. A. & Joosten, M. H. A. J. Two for all: receptor-associated kinases SOBIR1 and BAK1. *Trends Plant Sci*. **19**, 123–132 (2013). 10.1016/j.tplants.2013.10.003 24238702

[23] Ma X, Xu G, He P, Shan L (2016). SERKing co-receptors for receptors. Trends Plant Sci.

[24] Li J (2002). BAK1, an Arabidopsis LRR receptor-like protein kinase, interacts with BRI1 and modulates brassinosteroid signaling. Cell.

[25] Sun Y (2013). Structural basis for flg22-induced activation of the Arabidopsis FLS2-BAK1 immune complex. Science..

[26] Hu C (2018). A group of receptor kinases are essential for CLAVATA signalling to maintain stem cell homeostasis. Nat. Plants.

[27] Postma J (2016). Avr4 promotes Cf-4 receptor-like protein association with the BAK1/SERK3 receptor-like kinase to initiate receptor endocytosis and plant immunity. New Phytol.

[28] Singla B, Khurana JP, Khurana P (2009). Structural characterization and expression analysis of the SERK/SERL gene family in rice (*Oryza sativa*). Int J. Plant Genomics.

[29] Escocard de Azevedo Manhães AM, Ortiz-Morea FA, He P, Shan L (2021). Plant plasma membrane-resident receptors: surveillance for infections and coordination for growth and development. J Integr Plant Biol.

[30] Liu Q (2021). The calcium transporter ANNEXIN1 mediates cold‐induced calcium signaling and freezing tolerance in plants. EMBO J.

[31] Ding Y (2022). CPK28-NLP7 module integrates cold-induced Ca^2+^ signal and transcriptional reprogramming in. Arabidopsis. Sci. Adv..

[32] Liu Z (2017). Plasma membrane CRPK1-mediated phosphorylation of 14-3-3 proteins induce their nuclear import to fine-tune CBF signaling during cold Response. Mol. Cell.

[33] Yang T, Chaudhuri S, Yang L, Du L, Poovaiah BW (2010). A calcium/calmodulin-regulated member of the receptor-like kinase family confers cold tolerance in plants. J. Biol. Chem..

[34] Zhao C (2017). MAP kinase cascades regulate the cold response by modulating ICE1 protein stability. Dev. Cell.

[35] Zhang Z (2017). OsMAPK3 phosphorylates OsbHLH002/OsICE1 and inhibits its ubiquitination to activate *OsTPP1* and enhances rice chilling tolerance. Dev. Cell.

[36] Jia M (2022). Chilling-induced phosphorylation of IPA1 by OsSAPK6 activates chilling tolerance responses in rice. Cell Discov.

[37] Xu J (2010). Developing high throughput genotyped chromosome segment substitution lines based on population whole-genome re-sequencing in rice (*Oryza sativa* L.). BMC Genomics.

[38] Yan WH (2011). A major QTL, *Ghd8*, plays pleiotropic roles in regulating grain productivity, plant height, and heading date in rice. Mol. Plant.

[39] Tao Z (2011). *OsWRKY45* alleles play different roles in abscisic acid signalling and salt stress tolerance but similar roles in drought and cold tolerance in rice. J. Exp. Bot.

[40] Zhang Z (2017). Alternative functions of Hd1 in repressing or promoting heading are determined by Ghd7 status under long-day condition. Sci. Rep..

[41] Zhao Q (2018). Pan-genome analysis highlights the extent of genomic variation in cultivated and wild rice. Nat. Genet..

[42] Tajima F (1984). Statistical method for testing the neutral mutation hypothesis by DNA polymorphism. Genetics..

[43] Nielsen R (2005). Genomic scans for selective sweeps using SNP data. Genome Res..

[44] Gutaker RM (2020). Genomic history and ecology of the geographic spread of rice. Nat. Plants.

[45] Lee HK (2009). Drought stress-induced Rma1H1, a RING membrane-anchor E3 ubiquitin ligase homolog, regulates aquaporin levels via ubiquitination in transgenic Arabidopsis plants. Plant Cell.

[46] Rauniyar N (2015). Parallel reaction monitoring: A targeted experiment performed using high resolution and high mass accuracy mass spectrometry. Int. J. Mol. Sci..

[47] Santos, A. A., Carvalho, C. M., Florentino, L. H., Ramos, H. J. O. & Fontes, E. P. B. Conserved threonine residues within the A-loop of the receptor NIK differentially regulate the kinase function required for antiviral signaling. *PLoS One* **4**, (2009). 10.1371/journal.pone.0005781 PMC2686266 19492062

[48] Perraki A (2018). Phosphocode-dependent functional dichotomy of a common co-receptor in plant signalling. Nature.

[49] Xia C, Gong Y, Chong K, Xu Y (2021). Phosphatase OsPP2C27 directly dephosphorylates OsMAPK3 and OsbHLH002 to negatively regulate cold tolerance in rice. Plant Cell Environ..

[50] Komis, G. & Šamaj, J. Plant MAP kinases. *Methods Mol. Biol.*10.1007/978-1-4939-0922-3 (2014).

[51] Shi YY (2022). The power of selection: dissecting phenotypic plasticity and linkage drag underlying yield traits of rice (*Oryza sativa*) using selective introgression. Plant Breed.

[52] Alcázar R (2010). Natural variation at strubbelig receptor kinase 3 drives immune-triggered incompatibilities between *Arabidopsis thaliana* accessions. Nat. Genet..

[53] Chen C (2014). A two-locus interaction causes interspecific hybrid weakness in rice. Nat. Commun..

[54] Li B (2019). The receptor-like kinase NIK1 targets FLS2/BAK1 immune complex and inversely modulates antiviral and antibacterial immunity. Nat. Commun..

[55] Halter T (2014). The leucine-rich repeat receptor kinase BIR2 is a negative regulator of BAK1 in plant immunity. Curr. Biol..

[56] Hu B (2015). Variation in *NRT1.1B* contributes to nitrate-use divergence between rice subspecies. Nat. Genet..

[57] Chen X (2021). Protein kinases in plant responses to drought, salt, and cold stress. J. Integr. Plant Biol..

[58] Guo X (2023). Cold‐induced calreticulin OsCRT3 conformational changes promote OsCIPK7 binding and temperature sensing in rice. EMBO J.

[59] Zhang D (2019). OsCIPK7 point-mutation leads to conformation and kinase-activity change for sensing cold response. J. Integr. Plant Biol..

[60] Sun Y (2022). Plant receptor-like protein activation by a microbial glycoside hydrolase. Nature.

[61] Amorim-Silva V (2019). TTL proteins scaffold brassinosteroid signaling components at the plasma membrane to optimize signal transduction in Arabidopsis. Plant Cell.

[62] Lim R (2002). MADM, a novel adaptor protein that mediates phosphorylation of the 14-3-3 binding site of myeloid leukemia factor 1. J. Biol. Chem..

[63] Wang C (2017). OsCERK1-mediated chitin perception and immune signaling requires receptor-like cytoplasmic kinase 185 to activate an MAPK cascade in rice. Mol. Plant.

[64] Hu L (2015). The rice transcription factor WRKY53 suppresses herbivore-induced defenses by acting as a negative feedback modulator of mitogen-activated protein kinase activity. Plant Physiol.

[65] Liu D (2019). Identification of chilling tolerance of rice seedlings by cold water bath. Chin. Bull. Bot..

[66] Tamura K (2011). MEGA5: Molecular evolutionary genetics analysis using maximum likelihood, evolutionary distance, and maximum parsimony methods. Mol. Biol. Evol..

[67] Librado P, Rozas J (2009). DnaSP v5: A software for comprehensive analysis of DNA polymorphism data. Bioinformatics.

[68] Mansueto L (2017). Rice SNP-seek database update: new SNPs, indels, and queries. Nucleic Acids Res..

[69] Danecek P (2011). The variant call format and VCF tools. Bioinformatics.

[70] Tang Y (2020). OsNSUN2-mediated 5-methylcytosine mRNA modification enhances rice adaptation to high temperature. Dev. Cell.

