# Author Correction: The COG1-OsSERL2 complex senses cold to trigger signaling network for chilling tolerance in *japonica* rice

**DOI:** 10.1038/s41467-024-47895-0

**Published:** 2024-04-26

**Authors:** Changxuan Xia, Guohua Liang, Kang Chong, Yunyuan Xu

**Affiliations:** 1 The Key Laboratory of Plant Molecular Physiology, Institute of Botany, Chinese Academy of Sciences, Beijing, 100093 China; 2 University of Chinese Academy of Sciences, Beijing, 100049 China; 3 Jiangsu Key Laboratory of Crop Genetics and Physiology/Co-Innovation Centre for Modern Production Technology of Grain Crops, Key Laboratory of Plant Functional Genomics of the Ministry of Education, Yangzhou University, Yangzhou, 225009 China; 4 Present Address: Beijing Vegetable Research Center (BVRC), Beijing Academy of Agricultural and Forestry Sciences, Beijing, 100097 China

**Keywords:** Plant stress responses, Plant signalling, Plant domestication

Correction to: *Nature Communications* 10.1038/s41467-023-38860-4, published online 29 May 2023

In this article the affiliation details for Changxuan Xia, Kang Chong and Yunyuan Xu were incorrectly given as ‘University of the Chinese Academy of Sciences, Beijing 100049, China’ but should have been ‘University of Chinese Academy of Sciences, Beijing 100049, China’.

The original article has been corrected.

