# Supplementary material for: The COG1-OsSERL2 complex senses cold to trigger signaling network for chilling tolerance in japonica rice
Source: Nat Commun. 2023 May 29;14:3104. doi: 10.1038/s41467-023-38860-4 (PMC10227007; doi:10.1038/s41467-023-38860-4)
Supplement: Supplementary file 1 — Supplementary Information [file 41467_2023_38860_MOESM1_ESM.pdf]

# The COG1-OsSERL2 complex senses cold to trigger signaling network for chilling tolerance in *japonica* rice

Changxuan Xia[1, 2, #], Guohua Liang[3], Kang Chong[1, 2*], Yunyuan Xu[1, 2*]

1 The Key Laboratory of Plant Molecular Physiology, Institute of Botany, Chinese Academy of Sciences, Beijing, 100093, China.

2 University of the Chinese Academy of Sciences, Beijing 100049, China.

3 Jiangsu Key Laboratory of Crop Genetics and Physiology/Co-Innovation Centre for Modern Production Technology of Grain Crops, Key Laboratory of Plant Functional Genomics of the Ministry of Education, Yangzhou University, Yangzhou 225009, China

*Correspondence author: chongk@ibcas.ac.cn or xuyy@ibcas.ac.cn

#Present address: Beijing Vegetable Research Center (BVRC), Beijing Academy of Agricultural and Forestry Sciences, Beijing 100097, China.

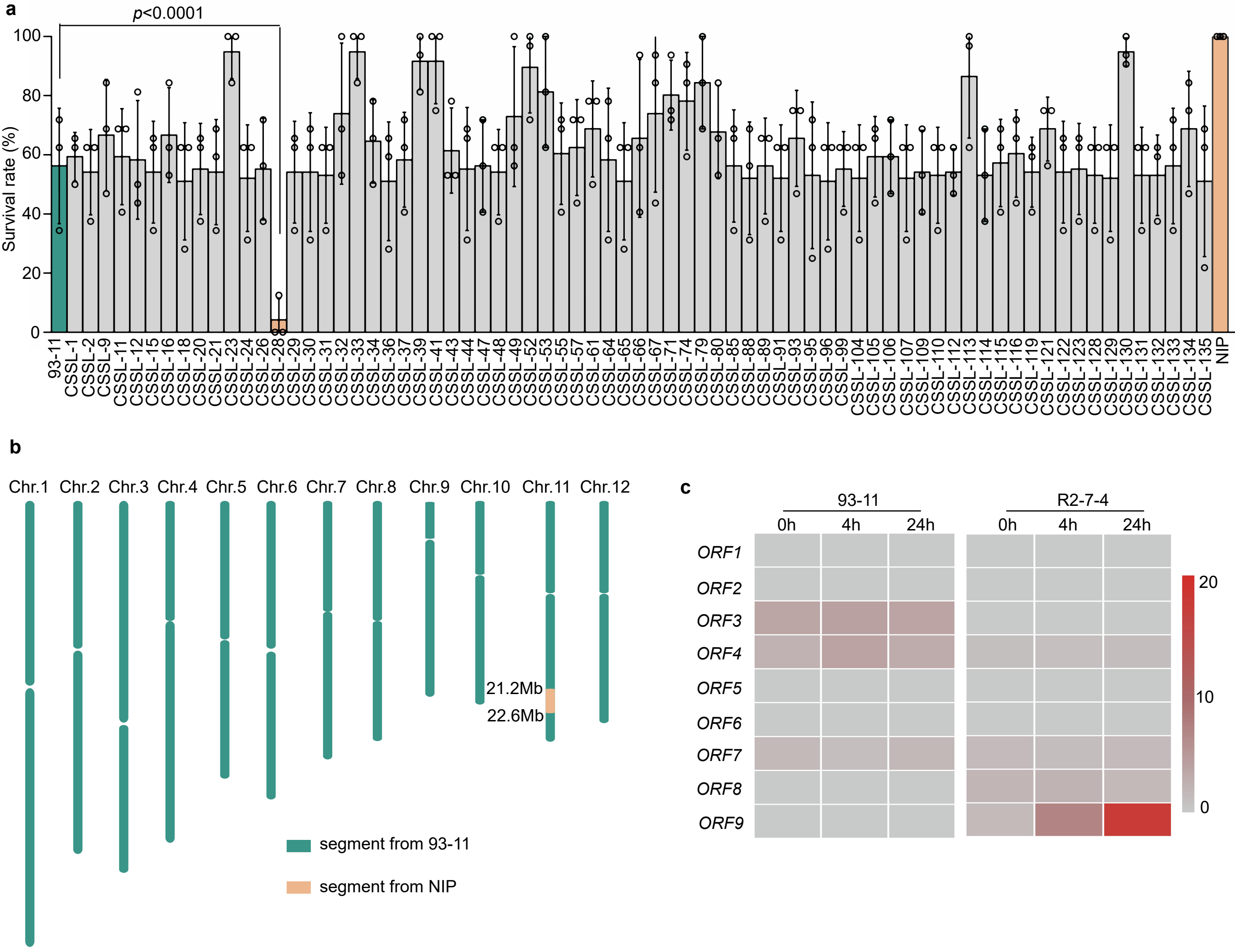


**Supplementary Figure 1│Chilling tolerance analysis of CSSLs and the expression levels of nine predicted genes in the *qCS11-jap* mapping interval, related to Figure 1.**

**a** Chilling tolerance of 72 individuals in the chromosome fragment substitution lines (CSSLs), the donor parent Nipponbare (NIP) and the recurrent parent 93-11. The survival rates were determined after recovery for 4-week. Data are means ± SD (two-tailed Student's *t*-test; n = 3 biological replicates). **b** Visualization of the R2-7-4 genotype. Green represents the chromosomal segment from 93-11 and yellow indicates segments from the NIP. **c** The expression levels of nine predicted genes in the *qCS11-jap* mapping interval in 93-11 and the homozygous recombinant line R2-7-4 after chilling treatment. The gray to red indicate FPKM values of 9 predicted genes varying from 0–20. Source data are provided as a Source Data file.

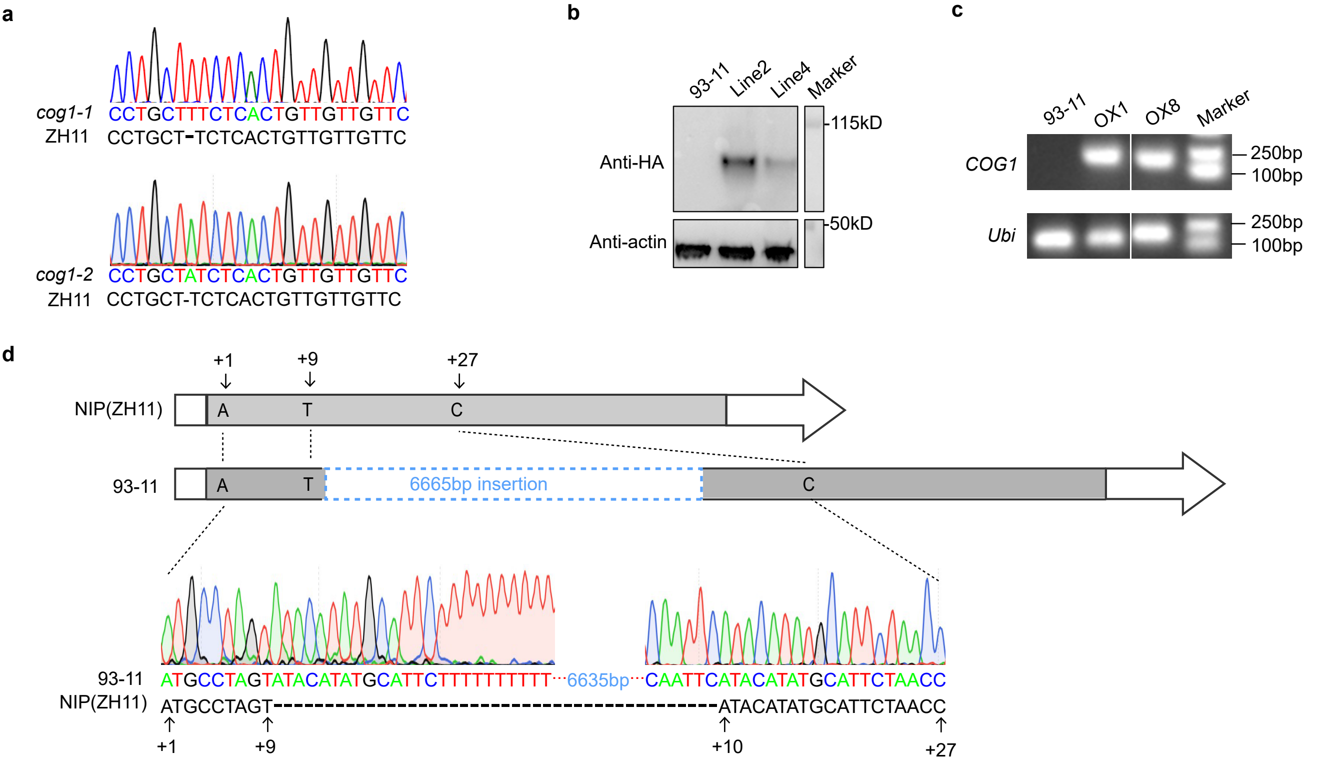


**Supplementary Figure 2│Identification of *COG1* mutants, *COG1* transgenic lines in 93-11 and the sequence comparison of *COG1* alleles between *japonica* (NIP and ZH11) and *indica* (93-11), related to Figure 2.**

**a** The sequencing results of mutations in *cog1-1* and *cog1-2*. **b** Identification of *COG1::COG1-HA* transgenic lines (Line2 and Line4) in 93-11 background via immunoblotting of HA-tag. Total protein extracts were used for immunoblotting with anti-HA and anti-actin antibodies. n = 3 biological replicates. **c** The identification of *COG1*-overexpression lines (OX1 and OX8) in 93-11 background with RT-PCR. n = 3 biological replicates. **d** The DNA sequence analysis of *COG1* alleles in 93-11, Nipponbare (NIP) and Zhonghua 11 (ZH11). Source data are provided as a Source Data file.

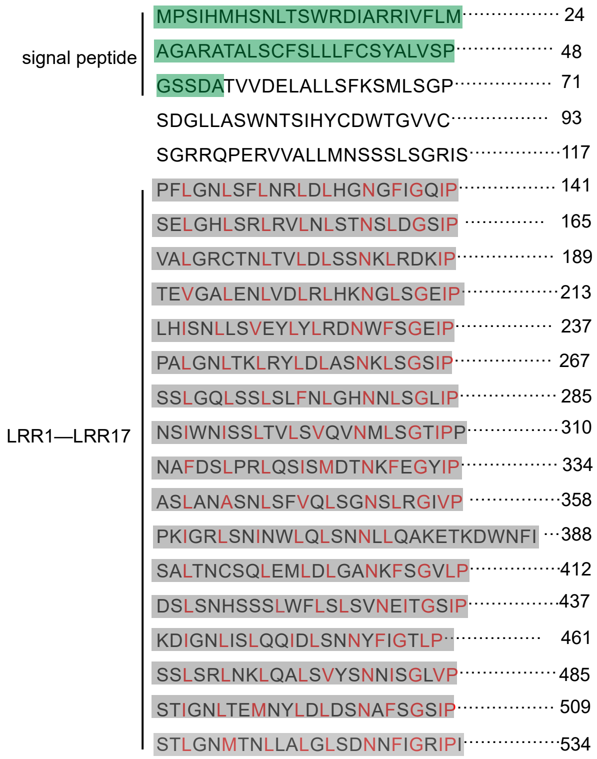


**Supplementary Figure 3│Analysis of the COG1 amino acid sequence, related to Figure 4.**

Analysis of the COG1 amino acid sequence. Residues 1 to 53 highlighted in green, comprised the signal peptide (SP). Residues 118 to 534 highlighted in gray, formed 17 leucine-rich repeat (LRR) domains. The conserved LRR-domain residues "LxxLxxLxNxLSGxIPxLGx" are showed in red.

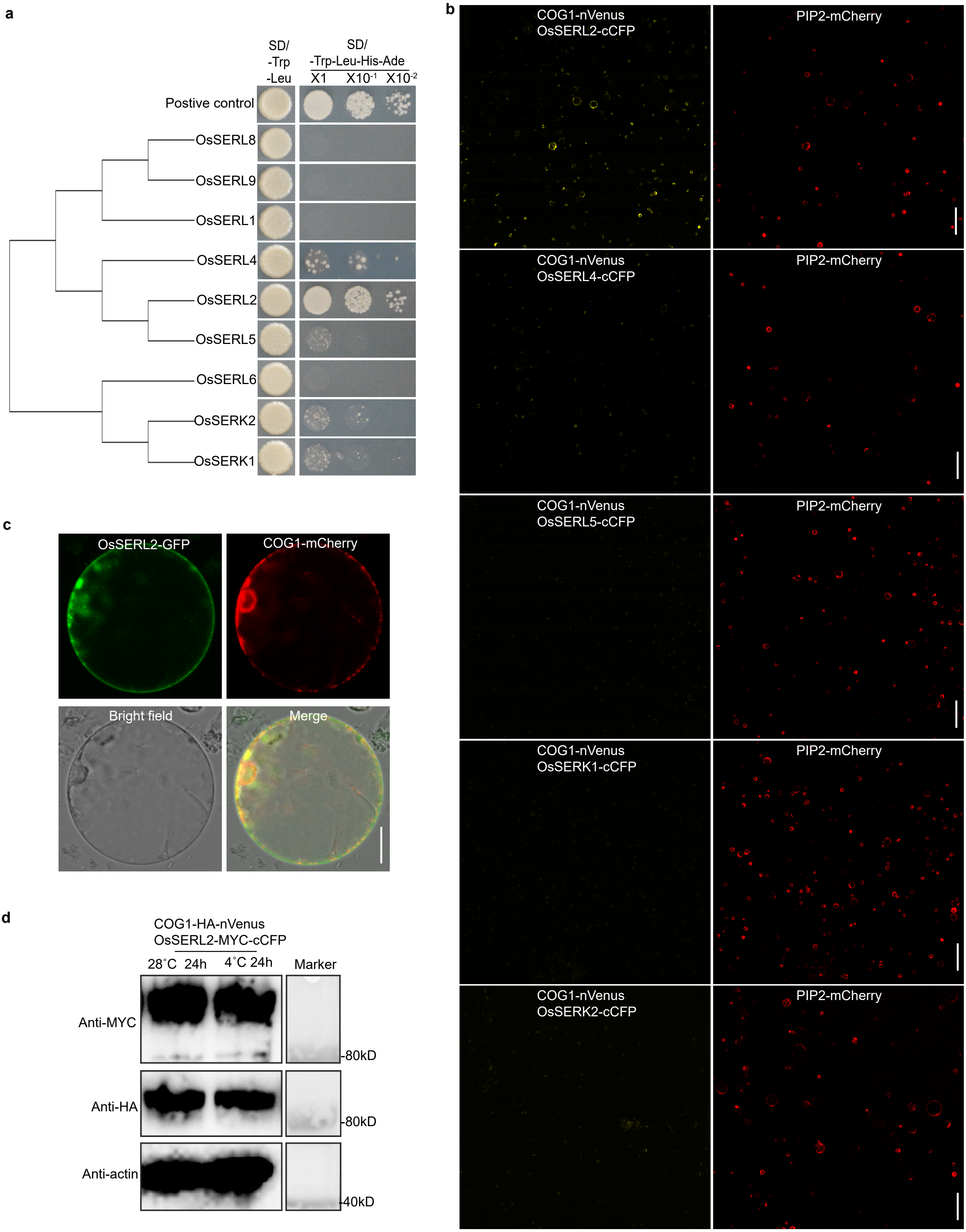


**Supplementary Figure 4 | Verification of the interaction between COG1 and OsSERL2, related to Figure 5.**

**a** Yeast two-hybrid assays were conducted to detect interactions between COG1 and SERK/SERL family members. Left, phylogenetic tree analysis showing relationships between SERK/SERL family members. Right, images of yeast colonies from the yeast two-hybrid assays. **b** BiFC assays were conducted in rice protoplasts to confirm the putative interactions between COG1 and some SERK/SERL family members. Scale bar=100 μm. **c** OsSERL2-GFP co-localizes with COG1-mCherry in rice protoplasts. Scale bar=10 μm. **d** Detection of the protein abundance of COG1-HA-nVenus and OsSERL2-MYC-cCFP in rice protoplasts at 28°C and 4°C treatment, related to Fig. 5c. Representative figures of b–d from three biological repeats. Source data are provided as a Source Data file.

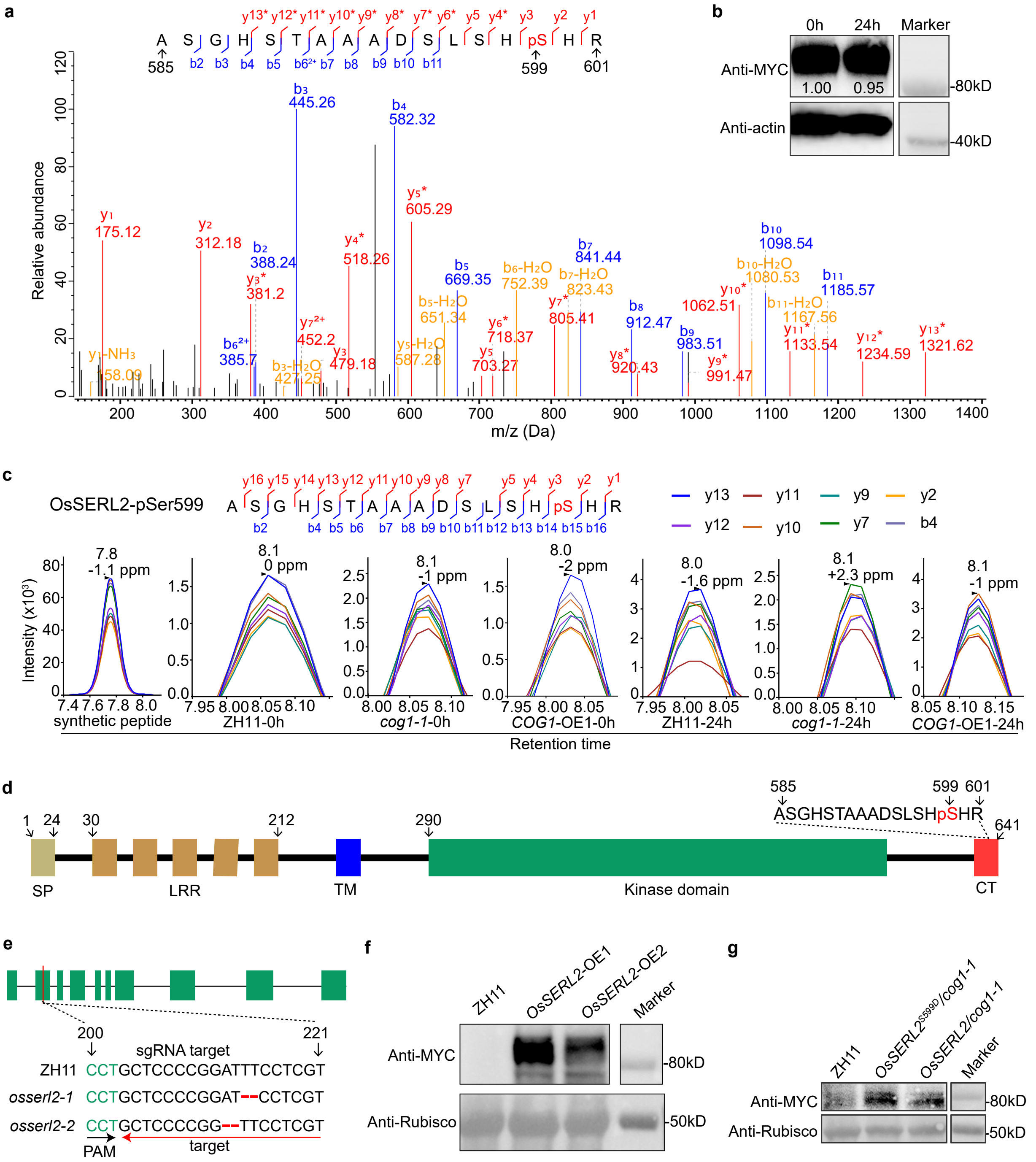

**Supplementary Figure 5│The chilling-induced phosphorylation of OsSERL2 at Ser599 was activated by COG1.**

**a** LC-MS/MS spectrum of a phosphopeptide (ASGHSTAAADSLSHpSHR) containing pSer599 of OsSERL2. Phosphoproteins were analyzed with TMT-labeled phosphorylation proteomics. The spectrum shows a collection of b, y ions produced by collision-induced dissociation of the intact peptide. Identified phospho-Ser was denoted as “pS”. The peptide sequence and fragmentation patterns are shown above the spectrum. **b** The protein level of OsSERL2-MYC in rice protoplasts expressing *OsSERL2::OsSERL2-MYC* after chilling treatment for 24 h **c** The fragment ions chromatogram of synthetic and endogenous OsSERL2-pSer599 phospho-peptide in ZH11, *cog1-1* and *COG1*-OE1 after treatment at 4°C for 24-hour as determined with LC-MS/MS using PRM method. Representative figures from three biological repeats. **d** Schematic of OsSERL2 structure. SP, signal peptide; LRR, leucine-rich repeat domain; TM, transmembrane domain; CT, C-terminal domain. The phosphorylated residue is indicated within the peptide (ASGHSTAAADSLSHpSHR) in red. **e** Identification of *osserl2-1* and *osserl2-2* mutants by sequencing. PAM, protospacer adjacent motif. **f** Identification of *MYC-OsSERL2* overexpression lines (*OsSERL2*-OE1 and OE2) by immunoblotting. Total protein extracts were used for immunoblotting with anti-HA and anti-Rubisco antibodies. n = 3 biological replicates. **g** Analysis of OsSERL2 levels in *35S::MYC-OsSERL2*$^{S599D}$*/cog1-1* (*OsSERL2*$^{S599D}$*/cog1-1*) and *35S::MYC-OsSERL2/cog1-1* (*OsSERL2/cog1-1*) plants by immunoblotting. Total protein extracts were used for immunoblotting with anti-HA and anti-Rubisco antibodies. n = 3 biological replicates. Source data are provided as a Source Data file.

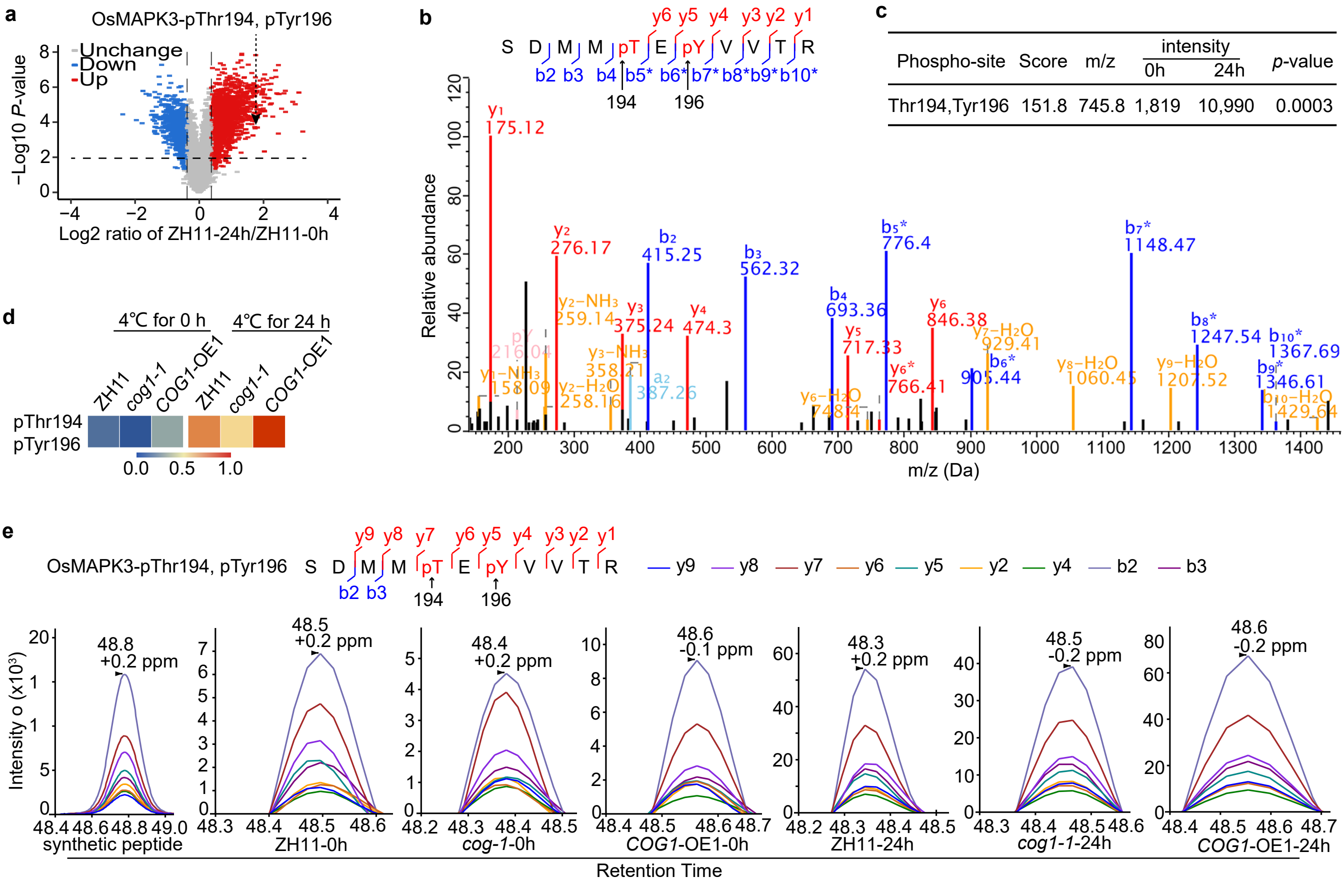


**Supplementary Figure 6│The chilling-induced phosphorylation levels of OsMAPK3 at Thr194 and Tyr196 were activated by COG1, related to Figure 7.**

**a** The volcano plot of TMT-labeled phosphorylation proteomics. The phosphorylation levels of OsMAPK3 at Thr194 and Tyr196 was induced after 24-hour chilling treatment. Data are shown as the means of three biological replicates (two-tailed Student's *t*-test). **b** LC-MS/MS spectrum of a phosphopeptide (SDMMpTEpYV-VTR) containing pThr194 and pTyr196 of OsMAPK3. Data are shown as the means of three biological replicates. **c** The phosphorylation intensity of OsMAPK3 at Thr194 and Tyr196 identified by TMT labeled phosphorylation proteomics after treatment at 4℃ for 24 h (two-tailed Student's *t*-test). **d** Quantification of pThr194 and pTyr196 levels on OsMAPK3 in ZH11, *cog1-1* and *COG1*-OE1 as identified with LC-MS/MS using the PRM method. The detailed data were shown in Supplementary Data 4. **e** The fragment ions chromatograms of synthetic and endogenous OsMAPK3 Thr194 and Tyr196 phospho-peptides in ZH11, *cog1-1* and *COG1*-OE1 after treatment at 4°C for 24-hour as identified with LC-MS/MS using the PRM method.

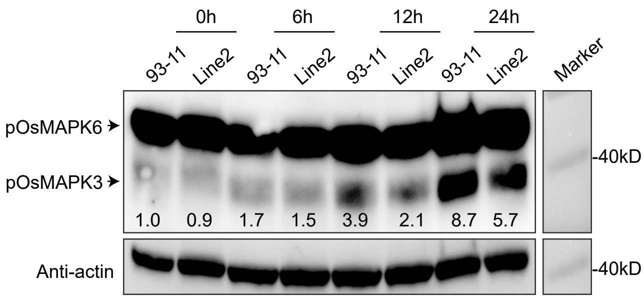


**Supplementary Figure 7│COG1 negatively regulates the activation of OsMAPK3 in 93-11 (*indica*).**

The phospho-Thr194 and phospho-Tyr196 levels of OsMAPK3 (pOsMAPK3) in 93-11 and *COG1::COG1-HA* transgenic line (Line2) in 93-11 background after treatment at 4℃ for 0, 6, 12 and 24 h. Total protein extracts were used for immunoblotting with anti-pTEpY and anti-actin antibodies. The actin protein level was used as the loading control. Representative figures from three biological repeats. Source data are provided as a Source Data file.
