# Supplementary material for: The COG1-OsSERL2 complex senses cold to trigger signaling network for chilling tolerance in japonica rice
Source: Nat Commun. 2023 May 29;14:3104. doi: 10.1038/s41467-023-38860-4 (PMC10227007; doi:10.1038/s41467-023-38860-4)
Supplement: Supplementary file 2 — Description of Additional Supplementary Files [file 41467_2023_38860_MOESM2_ESM.pdf]

## Description of Additional Supplementary Files

File Name: **Supplementary Data 1**

Description: Genotypes of 100 individuals and survival rate in the $F_2$ population.

File Name: **Supplementary Data 2**

Description: The summary of 67 representative rice accessions and the haplotype groups of *COG1*.

File Name: **Supplementary Data 3**

Description: Haplotype groups of *COG1* in 67 representative rice accessions.

File Name: **Supplementary Data 4**

Description: Chromatogram area of different fragment Ions for phospho-peptide of the OsSERL2 and OsMAPK3 in PRM analysis.

File Name: **Supplementary Data 5**

Description: Primer list, related to experimental procedures.

File Name: **Supplementary Data 6**

Description: All the identified phosphopeptides and its intensities of TMT labeled phosphorylation proteomics.
