# Supplementary material for: The COG1-OsSERL2 complex senses cold to trigger signaling network for chilling tolerance in japonica rice
Source: Nat Commun. 2023 May 29;14:3104. doi: 10.1038/s41467-023-38860-4 (PMC10227007; doi:10.1038/s41467-023-38860-4)
Supplement: Supplementary file 9 — Reporting Summary [file 41467_2023_38860_MOESM9_ESM.pdf]

nature portfolio

Corresponding author(s): Kang Chong

Last updated by author(s): Apr 19, 2023

Reporting Summary

Nature Portfolio wishes to improve the reproducibility of the work that we publish. This form provides structure for consistency and transparency in reporting. For further information on Nature Portfolio policies, see our Editorial Policies and the Editorial Policy Checklist.

Statistics

For all statistical analyses, confirm that the following items are present in the figure legend, table legend, main text, or Methods section.

n/a	Confirmed	
☐	☒	The exact sample size (*n*) for each experimental group/condition, given as a discrete number and unit of measurement
☐	☒	A statement on whether measurements were taken from distinct samples or whether the same sample was measured repeatedly
☐	☒	The statistical test(s) used AND whether they are one- or two-sided<br>*Only common tests should be described solely by name; describe more complex techniques in the Methods section.*
☒	☐	A description of all covariates tested
☒	☐	A description of any assumptions or corrections, such as tests of normality and adjustment for multiple comparisons
☐	☒	A full description of the statistical parameters including central tendency (e.g. means) or other basic estimates (e.g. regression coefficient) AND variation (e.g. standard deviation) or associated estimates of uncertainty (e.g. confidence intervals)
☐	☒	For null hypothesis testing, the test statistic (e.g. *F*, *t*, *r*) with confidence intervals, effect sizes, degrees of freedom and *P* value noted<br>*Give P values as exact values whenever suitable.*
☒	☐	For Bayesian analysis, information on the choice of priors and Markov chain Monte Carlo settings
☒	☐	For hierarchical and complex designs, identification of the appropriate level for tests and full reporting of outcomes
☒	☐	Estimates of effect sizes (e.g. Cohen's *d*, Pearson's *r*), indicating how they were calculated

Our web collection on statistics for biologists contains articles on many of the points above.

Software and code

Policy information about availability of computer code

Data collection	Leica TCS SP5 confocal microscope was used to capture images in BiFC assay;<br>CCD camera (Tanon-5200) were used to expose and capture images in western blot;<br>EASY-nLC 1000 UPLC system and Q ExactiveTM Plus (Thermo Fisher) coupled with chromatography system were used for Quantitative phosphoproteomics detection and Parallel reaction monitoring (PRM) quantitative phosphorylation detection;<br>Quant Studio3 (Thermo Fisher)) was used in qPCR .
Data analysis	MEGA 5.0 soft ware used in sequences aligning and phylogenetic tree construction;<br>DnaSP 5.1 ware used in s nucleotide diversity and Tajima's D analyses;<br>'SweeD' R package ware used to likelihood-based selective sweep detection;<br>Image J was used to analyze band intensity of pictures in western blot and angels of fluence response curves;<br>Skyline (v.3.6) was used to process MS data<br>GraphPad Prism 5 was used in two-side student's t-test;<br>Adobe Photoshop CS6 was used to cut images from western blot and confocal microscope;

For manuscripts utilizing custom algorithms or software that are central to the research but not yet described in published literature, software must be made available to editors and reviewers. We strongly encourage code deposition in a community repository (e.g. GitHub). See the Nature Portfolio guidelines for submitting code & software for further information.

Data

Policy information about availability of data

All manuscripts must include a data availability statement. This statement should provide the following information, where applicable:

- Accession codes, unique identifiers, or web links for publicly available datasets
- A description of any restrictions on data availability
- For clinical datasets or third party data, please ensure that the statement adheres to our policy

The mass spectrometry proteomics data have been deposited to the ProteomeXchange Consortium via the PRIDE [1] partner repository with the dataset identifier PXD042294. Data supporting the findings of this work are provided in the paper and its Supplementary Information file. Source data are provided with this paper.

Human research participants

Policy information about studies involving human research participants and Sex and Gender in Research.

Reporting on sex and gender	No sex and gender need to be considered in the study
Population characteristics	No population characteristics need to be considered in the study
Recruitment	No participants were recruited.
Ethics oversight	This study does not involve ethics insight

Note that full information on the approval of the study protocol must also be provided in the manuscript.

Field-specific reporting

Please select the one below that is the best fit for your research. If you are not sure, read the appropriate sections before making your selection.

☒ Life sciences ☐ Behavioural & social sciences ☐ Ecological, evolutionary & environmental sciences

For a reference copy of the document with all sections, see nature.com/documents/nr-reporting-summary-flat.pdf

Life sciences study design

All studies must disclose on these points even when the disclosure is negative.

Sample size	No statistical methods were used to predetermine sample size. For chilling treatment, at least 24 seedings were used. For protein extraction, immunoblotting and RNA isolation, at least six seedlings were collected for each sample to avoid individual differences.
Data exclusions	Unhealthy seedlings were excluded from the analyses.
Replication	All attempts at replication were successful
Randomization	The rice seeds were randomly selected and planted. And the seedlings with healthy growing status were used for each study.
Blinding	All the planted seedlings with healthy growing status were sampled or analyzed.

Reporting for specific materials, systems and methods

We require information from authors about some types of materials, experimental systems and methods used in many studies. Here, indicate whether each material, system or method listed is relevant to your study. If you are not sure if a list item applies to your research, read the appropriate section before selecting a response.

Materials & experimental systems

n/a	Involved in the study
[ ]	[x] Antibodies
[x]	[ ] Eukaryotic cell lines
[x]	[ ] Palaeontology and archaeology
[x]	[ ] Animals and other organisms
[x]	[ ] Clinical data
[x]	[ ] Dual use research of concern

Methods

n/a	Involved in the study
[x]	[ ] ChIP-seq
[x]	[ ] Flow cytometry
[x]	[ ] MRI-based neuroimaging

Antibodies

Antibodies used

anti- HA antibody (Sigma, H6908), anti-MYC antibody (CWBIO, CW0299), anti-Rubisco (Agrisera, AS10 700), anti-H+-ATPase (Agrisera,AS07 260), anti-BiP (YOUKE,YKZP054), anti-actin (huaxingbio, HX1833) anti-histone 3 (Sigma, 05-499) anti-His (Earthox, E022020), anti-pMBP (Sigma, 05-429) secondary antibody (anti-rabbit IgG, Cat# 7074, anti-mouse IgG, Cat# 7076, Cell Signaling Technology, dilution, 1:5,000)

Validation

For the commercial antibody, all validation statements can be found on the manufacturer's website.
